# Unavailability of experimental 3D structural data on protein folding dynamics and necessity for a new generation of structure prediction methods in this context

**DOI:** 10.1093/bioinformatics/btag020

**Published:** 2026-01-20

**Authors:** Aydin Wells, Khalique Newaz, Jennifer Morones, Jianlin Cheng, Tijana Milenković

**Affiliations:** Department of Computer Science and Engineering, University of Notre Dame, Notre Dame, IN 46556, United States; Institute for Computational Systems Biomedicine, University of Hamburg, 20148 Hamburg, Germany; Department of Computer Science and Engineering, University of Notre Dame, Notre Dame, IN 46556, United States; Department of Electrical Engineering and Computer Science, University of Missouri, Columbia, MO 65211, United States; Department of Computer Science and Engineering, University of Notre Dame, Notre Dame, IN 46556, United States

## Abstract

**Motivation:**

Protein folding is a dynamic process during which a protein’s amino acid sequence undergoes a series of 3D conformational changes en route to reaching a native 3D structure; these conformations are called folding intermediates. While data on native 3D structures are abundant, data on 3D structures of non-native intermediates remain sparse, due to limitations of current technologies for experimental determination of 3D structures. Yet, analyzing folding intermediates is crucial for understanding folding dynamics and misfolding-related diseases. Hence, we search the literature for available (experimentally and computationally obtained) 3D structural data on folding intermediates, organizing the data in a centralized resource. Also, we assess whether existing methods, designed for predicting native structures, can be utilized to predict structures of non-native intermediates.

**Results:**

Our literature search reveals six studies that provide 3D structural data on folding intermediates (two for post-translational and four for co-translational folding), each focused on a single protein, with 2–4 intermediates. Our assessment shows that an established method for predicting native structures, AlphaFold2, does not perform well for non-native intermediates in the context of co-translational folding; a recent study on post-translational folding concluded the same for even more existing methods. Yet, we identify in the literature recent pioneering methods designed explicitly to predict 3D structures of folding intermediates by incorporating intrinsic biophysical characteristics of folding dynamics, which show promise. This study assesses the current landscape and future directions of the field of 3D structural analysis of protein folding dynamics.

**Availability and implementation:**

https://github.com/Aywells/3Dpfi or https://academicweb.nd.edu/∼cone/3Dpfi/.

## 1 Introduction

Protein folding is a process by which a protein’s amino acid sequence folds into a 3D structure (or conformation). The 3D structure directs what other biomolecules the protein may interact with to carry out its function(s) (Gething and Sambrook 1992, Alberts *et al.* 2002). When a protein folds incorrectly, its misfolded 3D structure can disrupt normal cellular functions and contribute to diseases. Protein folding is a dynamic process, as a protein’s sequence gradually folds into a series of 3D structural conformations until arriving at a final, native structure (Privalov 1996, Wang *et al.* 2025); this time-series of 3D structural changes (including the native state) is called a *protein folding pathway*. The dynamics of the folding process can be studied from two prominent perspectives: *post-* and *co-translational* folding.

**Figure 1:**
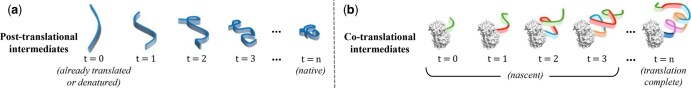
Illustrations of (a) post- and (b) co-translational protein folding pathway intermediates. (a) A protein’s *entire* sequence (blue line) undergoes conformational changes. The resulting 3D structures from time 0 to time *n* are the intermediates in the post-translational pathway. (b) A protein’s nascent sequence undergoes conformational changes as newly translated amino acids are added (additional line color) by the ribosome (grey entity) at time t=k to its already translated part from the previous time step (t=k−1), until all amino acids have been translated (t=n). The resulting 3D structures are the intermediates in the co-translational pathway. In both panels, the intermediates from time 0 to time n−1 are non-native, and the intermediate at time *n* is native. Note that the notion of an intermediate is tied to time, and not to the portion of sequence present in the 3D fold at time *t*: regardless of whether the 3D structure at time *t* is for the full sequence (panel a) or a subsequence (panel b), the structure is considered to be a (post- or co-translational, respectively) intermediate at time *t*. Also, note that both a post- and co-translational non-native intermediate can be *on-pathway*, meaning one that is traversed to reach the native state, or *off-pathway*, corresponding to a misfolded state that must be resolved before correct folding can continue (Baldwin 1996, Clark 2004). This means that “an intermediate” can be any (on- or off-pathway) intermediate on a (mis)folding pathway, rather than exclusively an intermediate with distinct biophysical characterizations such as local minima on the folding energy landscape. In our article, intermediates are whatever 3D structures are provided along a folding pathway in considered data.

Post-translational folding [aka folding *in vitro* (Duran-Romaña *et al.* 2025), folding in solution (Wang *et al.* 2025), or free folding (Tao and Xiao 2025)] is concerned with the structural changes of a protein’s *entire* sequence, which may be already translated (defined below) or instead may start from a denatured state (Basharov 2003). Specifically, a post-translational protein folding pathway is a time-series of 3D conformational changes that a protein’s full sequence undergoes until reaching a native state (Fig. 1a) (Basharov 2003, Englander and Mayne 2014). The full-sequence 3D conformations formed during this process (including the native structure) are called post-translational protein folding pathway intermediates (or just post-translational intermediates, for simplicity) (Plessa *et al.* 2021).

Co-translational folding is concerned with the structural changes of a protein’s *partial* sequence as it gradually grows *during* its translation by the ribosome. Namely, a co-translational protein folding pathway is a time-series of conformational changes that a protein’s sequence undergoes as newly translated amino acids are added to its already translated part from the previous time step, until all amino acids have been translated (Fig. 1b) (Nilsson *et al.* 2015, Moss *et al.* 2024, Wang *et al.* 2025). The 3D conformations corresponding to the gradually increasing subsequences formed during translation (including the native structure) are called co-translational protein folding pathway intermediates (or just co-translational intermediates) (Jacobs and Shakhnovich 2017).

A recent study summarizes (dis)advantages of post- versus co-translational folding (Duran-Romaña *et al.* 2025). Briefly, most of the understanding of protein folding comes from the former, with one of the most valuable insights being that a protein’s amino acid sequence encodes the information about its native 3D structure. However, even *in vitro*, a large fraction of the proteome cannot refold from a denatured state and instead tends to misfold and aggregate. Moreover, *in vivo*, an estimated one-third of the proteome in *Escherichia coli* (as a representative prokaryote) folds at least one entire domain co-translationally, and this fraction is likely even higher in eukaryotes given their slower translation rates. Moreover, slowing down or speeding up translation rates (e.g. as approximated by codon usage) can allow an already synthesized N-terminal portion more time to fold independently from a C-terminal portion or prevent specific folding intermediates from being populated, respectively. Consequently, the vectorial nature and translation rates are exploited *in vivo* to increase folding efficiency [i.e. how quickly a protein becomes functional (Wagner and Kiefhaber 1999, Siller *et al.* 2010, Tsytlonok and Itzhaki 2013)], which has implications for why many proteins fold more efficiently co-translationally than post-translationally (Duran-Romaña *et al.* 2025).

Analyzing folding intermediates is crucial for understanding protein folding dynamics and deepening insights on protein functions and misfolding-related diseases. Some data types—specifically kinetics and thermodynamics data—do exist on a somewhat large scale that aim to capture information on folding dynamics. For post-translational folding, databases with kinetics (protein folding rate) data exist for dozens to hundreds of proteins. For example, the Protein Folding DataBase (PFDB) contains kinetics (folding rate constant) data for 141 (89 two-state and 52 multi-state) single-domain globular proteins; a two-state protein folds via a two-state mechanism, directly from unfolded to folded, and a multi-state protein folds via a multi-state mechanism involving more than two intermediates. As of its publication in 2019, this made PFDB the largest available database of protein folding kinetics (Manavalan *et al.* 2019). As another example, Start2Fold contains kinetics (hydrogen/deuterium exchange) data extracted from the literature for 57 proteins with 219 residue sets (i.e. conformations), where each protein may have several residue sets categorized based on so-called protection levels (i.e. early, intermediate, and late for folding; and strong, medium, and weak for stability) (Pancsa *et al.* 2016). For post-translational folding, thermodynamics data also exist on a large scale. For example, ProThermDB contains thermodynamics (e.g. melting temperature and free energy) data for 31 580 (12 050 wild-type and 19 530 mutated) proteins (Nikam *et al.* 2021). For co-translational folding, we could not identify any organized database containing either kinetics or thermodynamics data. Instead, we could identify some isolated studies that provide data of these types for a handful of proteins (Section S1.1, available as supplementary data at *Bioinformatics* online).

Protein folding kinetics and thermodynamics data are valuable for understanding folding dynamics (Zhao 2013, Gelman and Gruebele 2014). However, both provide quantitative measures of the protein folding process, and typically, a quantity per protein rather than per intermediate of a protein. In other words, they lack 3D spatial resolution at the level of intermediates to reveal the atomic-level interactions and structural transitions that underpin the protein folding process. Similar holds for other types of data capturing properties of folding intermediates/pathways/dynamics that are obtained through HDX, site-resolved NMR spectroscopy, and mass spectrometry biophysical technologies (Englander and Mayne 1992, Narang *et al.* 2020). HDX probes changes in protein backbone exposure to infer which regions of the protein become transiently protected or exposed during folding. Site-resolved NMR provides residue-level information on local chemical environments and conformational exchange to detect partially folded states. Mass spectrometry, often coupled with HDX, detects mass shifts associated with hydrogen exchange, thus reports on the structural stability and kinetics of intermediate states.

To understand folding dynamics better, 3D structures of folding intermediates should be studied. Our goal is to provide a centralized resource discussing current data of this type. We comment on the availability of 3D structures of intermediates obtained (i) experimentally and (ii) computationally.

First, biophysical technologies such as NMR spectroscopy, cryo-EM, and X-ray crystallography (Klebe 2025) have contributed a wealth of experimentally determined 3D structures that are publicly available in the Protein Data Bank (PDB) (wwPDBconsortium 2019). These data are almost exclusively for native states of proteins rather than for non-native folding intermediates. This is because NMR and cryo-EM may capture instances of intermediate states, but their resolution or sensitivity may fall short when trying to detect a rapidly fluctuating sequence of conformational changes (Klebe 2025); and X-ray crystallography requires proteins to form crystals, a challenge that folding intermediates often cannot meet due to their unstable and dynamic nature (Zheng *et al.* 2015, Klebe 2025). Moreover, the protein folding process (folding of secondary and tertiary structures rather than necessarily the complete protein synthesis) often occurs in microseconds to seconds (Mayor *et al.* 2000, Duran-Romaña *et al.* 2025), making it difficult for the technologies to achieve the temporal resolution necessary to capture 3D structures of intermediates in real time (Freddolino *et al.* 2010, Lindorff-Larsen *et al.* 2011).

Second, the availability of large-scale experimentally derived data on native 3D structures has facilitated the development of computational approaches for predicting native 3D structures on an even larger scale (Outeiral *et al.* 2022, Abramson *et al.* 2024, Yuan *et al.* 2026). As we will show, there is a lack of large-scale experimentally determined 3D structural data on non-native folding intermediates. Given this, an interesting question arises: can computational approaches for native 3D structure prediction be used to predict 3D structures of non-native folding intermediates? For post-translational intermediates, this question was already asked recently, revealing a lack of success when using eight prominent approaches of this type, including AlphaFold2 (Outeiral *et al.* 2022). More recently, initial computational approaches have been introduced specifically for predicting 3D structures of post-translational intermediates (Huang *et al.* 2023, Zhao *et al.* 2023, 2024b); a recent review of these approaches is available (Zhao *et al.* 2024a). However, these valuable attempts have focused on *post-translational* intermediates. Our literature search done as a part of this study prior to the submission of our article has found no attempts focused on predicting 3D structures of *co-translational* intermediates. Hence, as a contribution of this study, we introduce an original research evaluation of whether AlphaFold2 can correctly predict experimentally determined 3D structures of co-translational intermediates that are currently available for a handful of proteins. Note that after the submission and during the revision of our article, two relevant studies were published that used an existing Monte Carlo approach and a molecular dynamics approach tailored to predict co-translational pathways for one and three proteins, respectively (Tao and Xiao 2025, Wang *et al.* 2025). However, none of them evaluated their predicted pathways by directly comparing 3D structures of their predicted co-translational intermediates against 3D structures of experimentally determined intermediates.

**Figure 2:**
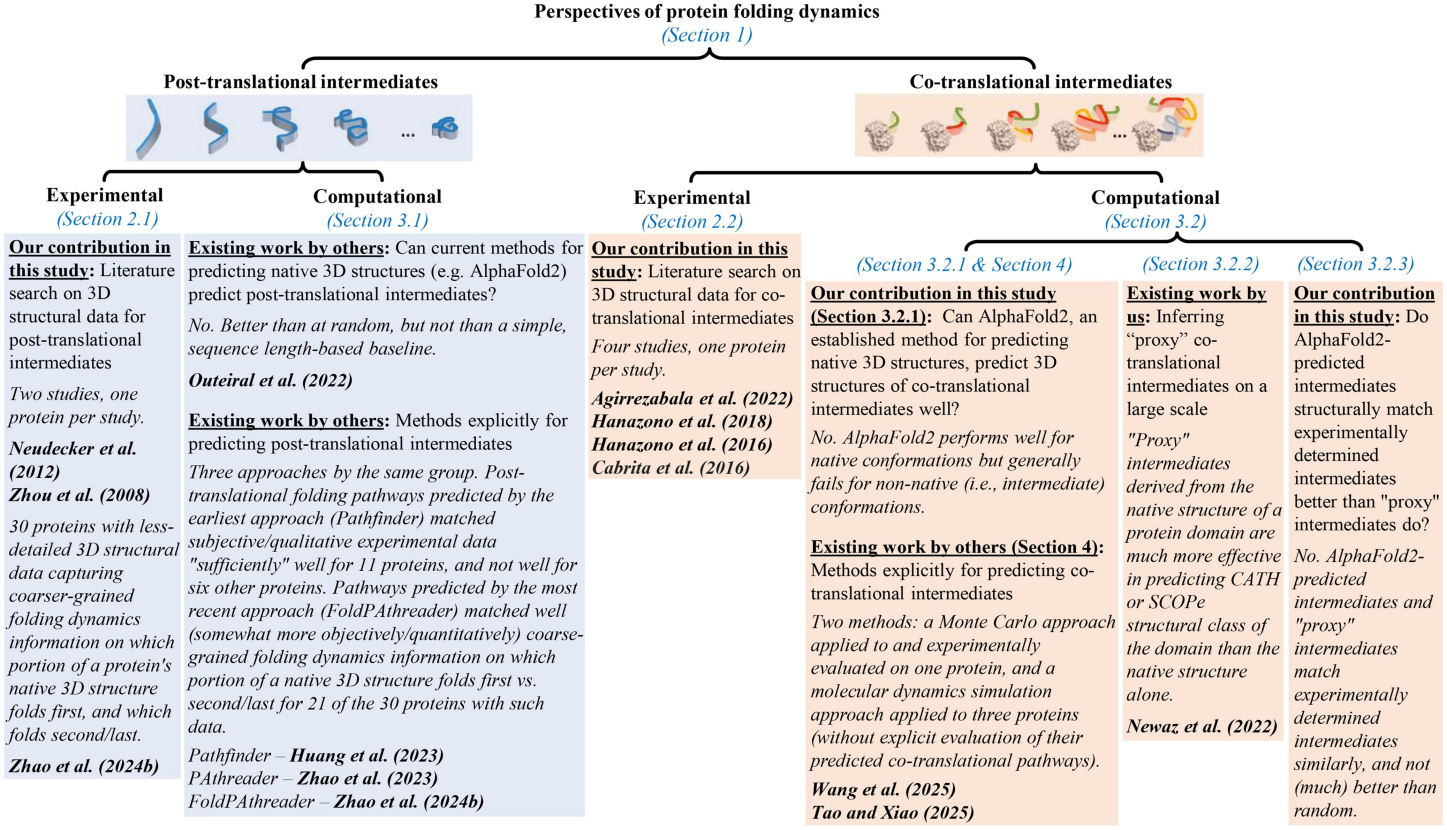
Summary of this article that is focused on availability and analysis of 3D structural data related to protein folding dynamics.

Unlike the existing work, in this article (Fig. 2), we search the literature for available experimentally determined (Section 2) and computational (Section 3) 3D structural data on folding intermediates, both post-translational (Sections 2.1 and 3.1) and co-translational (Sections 2.2 and 3.2) ones. Also, we investigate whether current native 3D structure predictors can predict well 3D structures of post-translational intermediates (from existing literature; Section 3.1) and co-translational intermediates (from our own analysis original to this article; Section 3.2). We aim to inform the community about available 3D structural data on folding intermediates and a (potential) need for a new generation of protein 3D structure predictors in this context. With our coverage of 3D structural data and structure prediction methods in the context of co-translational folding, we complement the above mentioned review that is on post-translational folding (Zhao *et al.* 2024a).

Other approach families for studying folding dynamics exist: molecular dynamics and Monte Carlo simulations, and coarse-grained models (Kmiecik *et al.* 2016). We touch on these in Section 3.1.

## 2 Experimental 3D structural data on protein folding pathway intermediates

### 2.1 Experimental post-translational intermediates

Our literature search on experimentally determined 3D structural data for post-translational intermediates identifies two studies (Zhou *et al.* 2008, Neudecker *et al.* 2012) that explicitly documented 3D structures of intermediates in PDB (Fig. S1 and Section S1.2, available as supplementary data at *Bioinformatics* online). Each of the two studies investigated one protein. The two proteins have sequence lengths of on the order of 100 amino acids. Each study reported two post-translational intermediates per protein—one pre-native and one native. For each intermediate, a deposited sequence range (i.e. the amino acid sequence that is reported) and a modeled sequence range (i.e. the portion of the sequence that has available 3D structure information) were reported in PDB; note that the two can differ.

While these two studies have successfully resolved 3D structures of intermediates, they are limited in scope—the number of proteins studied (only two), the size of proteins studied (quite short), and the number of intermediates reported (only two). As the current 3D structural data on post-translational intermediates is sparse, owing to the limitations of biophysical technological discussed above, there is a need for novel technologies for this purpose.

For each study’s protein, we examine the 3D structural similarity between the given protein’s first intermediate and its second intermediate, in terms of the Template Modeling score (TM-score) (Zhang and Skolnick 2004). TM-scores range between 0 and 1, where scores below 0.17 indicate random-like similarities, scores above 0.3 indicate significant similarities, and scores above 0.5 indicate the same overall fold (Section S2.1, available as supplementary data at *Bioinformatics* online). We find that the TM-score between the two intermediates is reasonably high, between 0.77 and 0.80, depending on the protein (Fig. S1, available as supplementary data at *Bioinformatics* online). Also, for completeness, we examine each study’s aim in more detail; due to space constraints, we report this in Section S1.2, available as supplementary data at *Bioinformatics* online.

Note that the recent initial studies on computational prediction of post-translational folding pathways (mentioned in Section 1 and discussed in Section 3.1) contain less-detailed 3D structural data capturing coarser-grained folding dynamics information for 30 proteins (Zhao *et al.* 2024a, 2024b). Here, the only knowledge on folding dynamics is which portion of a protein’s *native* 3D structure folds first, and which folds second/last; information is available only for these two temporal states (Zhao *et al.* 2024a,b).

### 2.2 Experimental co-translational intermediates

**Figure 3:**
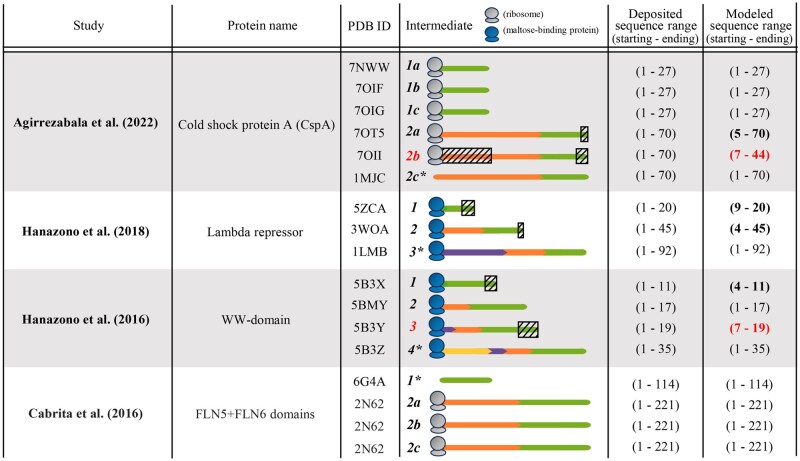
Details of the four studies we have identified that report 3D structural data of co-translational intermediates. The protein from the first study listed has intermediates for two deposited sequence ranges (1: 1–27, 2: 1–70). For each of the two intermediates, three distinct conformations are provided (a, b, and c). Conformation 2c that is no longer attached to the ribosome is the native structure of the protein (as denoted by an asterisk); conformations 2a and 2b have the same deposited sequence range as conformation 2c, but have structural differences to conformation 2c due to the appearance of the ribosome. Similar holds for the last study listed, whose protein has intermediates corresponding to two sequence ranges (1: 1–114, 2: 1–221). Intermediate 1 corresponds to a native structure of isolated (i.e. post-translationally folded, ribosome-independent) sequence region shown in green (corresponding to domain FLN5). Intermediate 2 corresponds to co-translationally folded sequence consisting of domains FLN5 (green) and FLN6 (orange) combined. For intermediate 2, three distinct confirmations are provided (a, b, and c; note that 3D structures of all three of these are available in the same PDB file, with the same PDB ID 2N62). For more information on why the shorter of the two intermediates is the native structure, see the last paragraph in Section S1.3, available as supplementary data at *Bioinformatics* online. In the second and third studies listed, the given protein has three (1–3) and four (1–4) intermediates, respectively, of growing length, each with a single conformation. For both studies, the last intermediate is the native structure. The third column contains the PDB ID of each conformation of an intermediate. In the fourth column, each conformation is represented as a colored line where colors denote the same sequence region between intermediates. Also in the fourth column, the gray or blue entity at the C-terminus is a ribosome or maltose-binding protein, respectively; only the conformations with PDB IDs 1MJC and 6G4A do not have such an entity attached to them because these intermediates were experimentally studied in the absence of either entity. The fifth and sixth columns are defined in the same way as in Fig. S1, available as supplementary data at *Bioinformatics* online. A modeled sequence range is bolded if a portion of the deposited sequence is missing from the modeled sequence; such a discrepant subsequence is represented with a hatched box overlaid on the deposited sequence in the fourth column (Section S2.1, available as supplementary data at *Bioinformatics* online, discusses how we handle 3D structural comparison in Section 3.2 and later in the presence of minor subsequence discrepancies). A modeled sequence range is in red if its corresponding conformation is excluded from subsequent analyses in Section 3.2 and later because of major subsequence discrepancies considered to affect data completeness/quality (described in Section 2.2).

Our literature search on experimentally determined 3D structural data for co-translational intermediates identifies only four studies (Cabrita *et al.* 2016, Hanazono *et al.* 2018, 2016, Agirrezabala *et al.* 2022) that explicitly document 3D structures of intermediates in PDB (Fig. 3 and Section S1.3, available as supplementary data at *Bioinformatics* online). The current data on co-translational intermediates suffer from similar limitations as the data on post-translational intermediates—each of the four studies on co-translational intermediates also reports a single protein, the studied proteins are also relatively short (all but one of the four studies analyze proteins with fewer than 100 residues), all four studies also provide quite few (2–4) intermediates. So, the current data on co-translational intermediates are also sparse. This further emphasizes the need for innovative experimental biophysical technologies for better capturing data on intermediates.

Across the four studies/proteins, there are 11 distinct intermediates. Just as multiple 3D structural conformations may be reported for a native state, multiple conformations may be reported for an intermediate state as well. For three out of the 11 intermediates, three conformations have been reported per intermediate; for the remaining eight intermediates, only one conformation has been reported per intermediate (Fig. 3). Hence, there are a total of 17 conformations for the 11 intermediates. For six of the conformations, their modeled sequences are shorter than their deposited sequences (PDB IDs 70T5, 70II, 5B3X, 5B3Y, 5ZCA, 3W0A). Of the six, we discard from further analyses the conformation of an intermediate with PDB ID 70II due to a huge data loss of ∼50% in the modeled sequence compared to the deposited sequence. Also, we discard from further analyses the intermediate with PDB ID 5B3Y, because (i) this is the second largest data loss, (ii) this intermediate’s deposited sequence adds only two extra amino acids (deposited sequence range of 1–19) to the intermediate with PDB ID 5BMY (deposited sequence range of 1–17), and (iii) 5B3Y loses many of its amino acids in the modeled sequence but 5BMY does not lose any; hence, 5BMY is an intermediate of much higher data quality, and again, almost as long as the deposited sequence of 5B3Y. We continue to analyze the remaining four conformations (i.e. 70T5, 5B3X, 5ZCA, 3W0A) with some minor data loss. In total, we proceed with analyzing 15 conformations for 10 intermediates (i.e., all intermediates from Fig. 3 except the two with PDB IDs 70II and 5B3Y).

We measure structural similarities (TM-scores) between all pairs of (partial and full) conformations of intermediates of the same protein (Section S2.1, available as supplementary data at *Bioinformatics* online). We find the following (Fig. S2, available as supplementary data at *Bioinformatics* online). First, we focus on distinct conformations for the same intermediate (e.g. 2a versus 2b versus 2c from a given study). All corresponding TM-scores are below 0.5, indicating that no two conformations of the same intermediate have the same fold. Second, we focus on the conformational change of a sequence of an intermediate gradually over time (e.g. a green sequence in an intermediate versus the same green sequence in the next intermediate for the same protein). Overall, we observe quite a large conformational change of the same sequence between consecutive time points in the presence of additional amino acids being translated and added to the 3D structure, often resulting in a changed fold (i.e. TM-score below 0.5) of the given sequence during translation. Third, to evaluate the effect of time passed, we compare conformations corresponding to the same sequence of an intermediate at closer versus more distant times (e.g. a sequence from time 1 to time 2 versus the same sequence from time 1 to time 3). In all comparisons, more time-distant conformations of intermediates tend to show higher levels of conformational change than time-closer conformations for the same protein. For more detailed results, see Section S3.1, available as supplementary data at *Bioinformatics* online.

For completeness, we summarize the aims of the four studies from this section in more detail. Due to space constraints, we report this in Section S1.3, available as supplementary data at *Bioinformatics* online.

## 3 Computational 3D structural data on protein folding pathway intermediates

### 3.1 Computational post-translational intermediates

With advancements in protein 3D structure prediction, available data on native structures has increased drastically. Prior to the release of the AlphaFold database, ∼200 000 experimentally determined 3D structures were reported in PDB—just a fraction of the billions of known protein sequences (Varadi *et al.* 2024). Now, computationally predicted 3D structures are available for over 214 million proteins (Varadi *et al.* 2024). Given the lack of experimentally determined 3D structural data on folding intermediates, can existing methods for predicting native 3D structures accurately predict 3D structures of intermediates?

Outeiral *et al.* (2022) investigated this question for eight prominent existing methods, including AlphaFold2, on *post-translational* intermediates. Each of 170 analyzed proteins was assigned one of two folding kinetics classes (Pancsa *et al.* 2016, Manavalan *et al.* 2019): two-state (90/170) or multi-state (80/170). Seventy-nine of the 90 two-state proteins had additional kinetics data—folding rate constants. To predict a protein’s folding pathway, the existing methods were used to output intermediate 3D structures produced during the process of predicting the native structure (Outeiral *et al.* 2022). The seven methods (except AlphaFold2) were used to predict 10 pathways per protein. Five of the more scalable methods were also used to predict 200 pathways per protein. For AlphaFold2, only one predicted pathway was available per protein. We could not find information on the number of intermediates per pathway, nor the predicted structures of the intermediates, reported in the article by Outeiral *et al.* (2022).

When evaluating a method’s predicted pathway, Outeiral *et al.* (2022) simply assumed that many proteins fold by first forming secondary structures, followed by forming tertiary contacts between them. So, a predicted pathway was examined using its predicted 3D structural intermediates to track when native contacts formed between each pair of secondary-structure elements over time. If all pairs formed their native contacts around the same time, this indicated a two-state folding mechanism, where folding occurs in a single concerted step. In contrast, if some pairs formed contacts earlier and others later, this suggested a multi-state folding mechanism. This information was then used to evaluate whether a method’s predicted pathways (i) are predictive of a protein’s folding kinetics class (i.e. two-state or multi-state), and (ii) correlate with experimentally measured folding rate constants.

For the first evaluation task, all structure prediction methods achieved statistically significant yet quite modest performance (with the area under the receiver-operating characteristic curve, i.e. AUROC, scores between 0.56 and 0.675, when predicting 10 pathways per method per protein). The methods were compared to a trivial baseline relying only on chain length; chain length served as a deliberately simple, sequence-agnostic baseline to test whether structure prediction methods actually learned meaningful folding physics. This simple baseline outperformed all structure prediction methods (AUROC score of 0.739). Results in the first evaluation task were qualitatively similar for the other performance accuracy measures and when predicting 200 pathways per method per protein (Section S2.2 and Table S1, available as supplementary data at *Bioinformatics* online). For the second evaluation task, chain length had the strongest and correctly-signed correlation, again outperforming any structure prediction method (Section S2.2 and Fig. S3, available as supplementary data at *Bioinformatics* online).

**Table 1:** Structural similarity (with respect to TM-score) of experimentally determined (exp.) versus “proxy” versus AlphaFold2-predicted co-translational intermediates.^a^

Study	PDB ID	Exp. intermediate	TM-score of “proxy” intermediate versus exp. intermediate	TM-score of AlphaFold2 intermediate versus exp. intermediate	TM-score of “proxy” intermediate versus AlphaFold2 intermediate
Hanazono *et al.* (2018)	5ZCA	1	0.22	0.41	**0.85**
	3WOA	2	0.27	**0.53**	**0.59**
Hanazono *et al.* (2016)	5B3X	1	0.43	0.28	0.33
	5BMY	2	0.46	0.21	0.29

a For reasons stated in the text, this analysis is done on two of the four considered studies/proteins: Hanazono *et al.* (2018) and Hanazono *et al.* (2016). For each study/protein, results are shown for all (both) of its *non-native* intermediates. All TM-scores with values higher than 0.50 are bolded, corresponding to structures that have the same overall fold.

These findings suggested that folding pathways predicted by the existing structure prediction methods fail to meaningfully capture folding kinetics data or correlate with experimental folding rate data. In other words, the existing methods fail to accurately model post-translational folding pathways.

The existing methods, originally designed to predict native 3D structures, were applied by Outeiral *et al.* (2022) in a naive way to generate pathways that yielded the native structure, without any direct capability to capture protein folding dynamics. Instead, the recent Pathfinder (Huang *et al.* 2023) is a pioneering method explicitly designed to predict a post-translational folding pathway, i.e. to capture the dynamics of post-translational folding; it is a Monte Carlo approach that uses conformational sampling to explore the transition probabilities of folding intermediates and infer likely folding pathways. To assess whether a protein’s post-translational pathway predicted by Pathfinder captured folding dynamics well, the contact order (average sequence distance between residues that form native contacts) was computed in each intermediate of the pathway. Then, by *qualitatively* analyzing how contact order evolved over the course of the predicted pathway and comparing this information to experimentally observed folding data, it was evaluated whether the predicted pathway “sufficiently” captured realistic folding dynamics (“sufficiently” because the evaluation was qualitative/subjective, based on descriptive folding information from the literature, rather than quantitative/objective). Pathfinder predicted folding pathways “sufficiently” well for 11 proteins, while it did not do well for six other proteins (Huang *et al.* 2023).

In parallel to Pathfinder, the same lab proposed PAthreader (Zhao *et al.* 2023), which is based on remote homologous template recognition. Their more recent approach, FoldPAthreader (Zhao *et al.* 2024b), combines the ideas of Pathfinder and PAthreader. When evaluated on the 30 proteins with only coarse-grained folding dynamics information on which portion of the *native* 3D structure folds first versus second/last (Section 2.1), for 21 of them, FoldPAthreader’s predicted post-translational pathways were judged as being consistent with that information.

Monte Carlo approaches such as Pathfinder complement other common computational strategies such as molecular dynamics simulations (which provide atomistic detail through force field-driven trajectories) and coarse-grained models (which simplify molecular representations to enable broader exploration of folding behavior) (Kmiecik *et al.* 2016). Despite their differences, all three approach types share limitations, specifically a reliance on highly parameterized energy functions, simplified assumptions (e.g. modeling only backbone or only side-chain atoms, or grouping several atoms into a single unit), or constrained timescales, which can introduce significant biases in predictions of protein folding dynamics and a limited simulated timescale (Kmiecik *et al.* 2016, Hu *et al.* 2017, Gershenson *et al.* 2020). Moreover, the existing approaches, including those from the Outeiral *et al.* (2022) study as well as the Pathfinder approach from the Huang *et al.* (2023) study, rely on experimental observations of secondary-structure formation or folding kinetics information for validation—rather than direct comparison to 3D structures of intermediates (as such data do not exist for post-translational intermediates other than the two studies mentioned in Section 2.1). Same holds for the FoldPAthreader approach. And while its evaluation did get somewhat less qualitative/subjective and more quantitative/objective, still only coarse-grained 3D structural data capturing limited folding dynamics information on top of the native structures was used (Zhao *et al.* 2024b). We imagine that having actual 3D structures of post-translational intermediates along a folding pathway could help better evaluate and refine post-translational folding pathway prediction tools.

### 3.2 Computational co-translational intermediates

#### 3.2.1 Can AlphaFold2, an established method for predicting native 3D structures, predict 3D structures of co-translational intermediates?

Despite significant advances in protein structure prediction, our literature search has uncovered no computational methods for predicting 3D structures of co-translational intermediates—with the exception of two studies (Tao and Xiao 2025, Wang *et al.* 2025) published after submission of this article of ours. So, as an original contribution to this article, we explore whether AlphaFold2, a prominent existing method designed for prediction of native 3D structures, can predict 3D structures of co-translational intermediates—following a similar question that Outeiral *et al.* (2022) investigated for post-translational intermediates. While the studies from Section 3.1 evaluated predicted post-translational intermediates with respect to quantitative measures (e.g. folding kinetics data) or qualitative information (e.g. secondary-structure formation information), we cannot do the same for predicted co-translational intermediates, because such data are limited for co-translational intermediates (Section 1). Instead, we predict 3D structures of co-translational intermediates for the four proteins that have experimentally determined 3D structures of co-translational intermediates available (Section 2.2). Then, we evaluate the predictions by directly comparing them to the 3D structures of experimentally determined co-translational intermediates. In this analysis, we rely on the 15 considered conformations for the 10 intermediates, which we have already cleaned to remove data noise (Section 2.2), hence hopefully yielding higher-confidence results from our analyses. We predict 3D structures of the co-translational intermediates as follows.

For each of the 15 conformations of the intermediates, we give its deposited sequence (excluding the ribosome or maltose-binding protein) as input into AlphaFold2, specifically ColabFold v1.5.2 (Mirdita *et al.* 2022). As a result, each conformation has a corresponding AlphaFold2-predicted 3D structure. AlphaFold2 generates five predicted structures for each sequence, ranked by confidence. We report results for the top-ranked predicted structure. Nonetheless, results for all five AlphaFold2-predicted structures of a given sequence are qualitatively similar and quantitatively almost identical regardless of which AlphaFold2-predicted structure for a given sequence is used (Fig. S4, available as supplementary data at *Bioinformatics* online). Note that we use AlphaFold2 rather than AlphaFold3 because the main improvements of the latter compared to the former focus on modeling protein complexes and biomolecular interactions, rather than offering tremendously significant advances for predicting the native structure of a single-chain protein (Abramson *et al.* 2024, Elofsson 2026).

**Figure 4:**
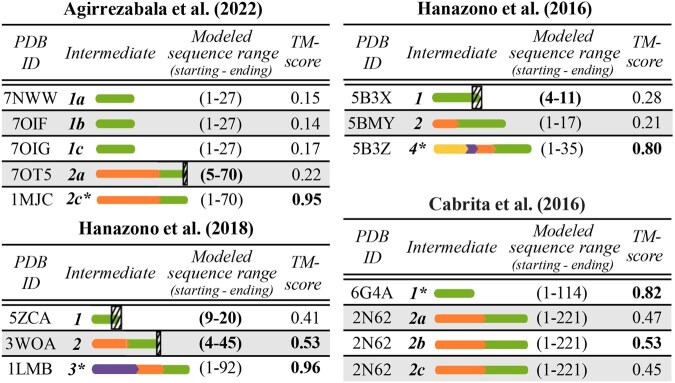
Structural similarities in terms of TM-scores between the AlphaFold2-predicted versus experimentally determined structures for the 15 considered conformations of the 10 co-translational intermediates. Each of the four studies, has a corresponding table; the first three table columns are already explained in Fig. 3 (except that here we do not show or analyze an attached entity at the C-terminus). The fourth table column reports the TM-score between an AlphaFold2’s prediction and the corresponding experimentally determined confirmation; all TM-scores above 0.50 are bolded, indicating the same overall fold. 3D visualizations of the AlphaFold2-predicted structures are shown in Fig. S5, available as supplementary data at *Bioinformatics* online. Note that each result here is for the highest-ranked AlphaFold2-predicted structure; results for all five highest-ranked AlphaFold2-predicted structures are shown in Fig. S4, available as supplementary data at *Bioinformatics* online.

To evaluate how well AlphaFold2 predicts 3D structures of co-translational intermediates, for each conformation, we compare its experimentally determined structure to its AlphaFold2-predicted structure using TM-score (Section S2.1, available as supplementary data at *Bioinformatics* online). Our findings are as follows (Fig. 4). Unsurprisingly, AlphaFold2 performs well for native conformations of intermediates (TM-scores in the 0.80–0.96 range, depending on the protein). There is a stark contrast in its performance for non-native intermediates, where it does not work as well. Namely, of the 15 conformations of the intermediates that are evaluated, 11 are non-native. For nine of these, AlphaFold2 yields TM-scores below 0.5, specifically in the 0.14–0.47 range (indicating different folds), and for the remaining two non-native conformations, AlphaFold2 yields TM-scores of just above 0.5. Due to space constraints, for key observations per study, see Section S3.2, available as supplementary data at *Bioinformatics* online.

Our findings reinforce that current computational structure prediction methods (like AlphaFold2) that are designed to predict native 3D structures are not inherently able to capture well co-translational intermediates. This observation is consistent with the previous findings for post-translational intermediates (Outeiral *et al.* 2022) (Section 3.1).

#### 3.2.2 Inferring “proxy” co-translational intermediates

Computational analyses of *post-translational* folding have been done on at least somewhat large scale for dozens to hundreds of proteins—though on non-3D structural data (Section 3.1). For *co-translational* folding, we could only analyze four proteins for which 3D structures of their intermediates are available, since no quantitative nor qualitative data exist on co-translational intermediates (Section 3.2.1). This raises the question of whether 3D structures of co-translational intermediates can be predicted on a larger scale, and if so, how they would be validated. Our analysis in Section 3.2.1 reveals that one of the best existing methods designed to predict native 3D structures—AlphaFold2—is not equipped to capture structures of co-translational intermediates. Certainly, testing other method types (Huang *et al.* 2023, Zhao *et al.* 2024b, Tao and Xiao 2025, Wang *et al.* 2025) in the task of predicting 3D structures of co-translational intermediates could be a valuable future direction, although validation would be challenging given the lack of data on co-translational intermediates. So, how may it be possible to predict and validate co-translational intermediates on a much larger scale? In our recent work, we took a step forward in this direction, by utilizing a bulk of data that is readily available—native 3D structures—to infer “proxy” co-translational intermediates on a large scale (Newaz *et al.* 2022).

Briefly, we extracted a protein’s 3D structural “proxy” co-translational intermediates from its native structure as follows (Newaz *et al.* 2022). Motivated by the co-translation process (Fig. 1b), the protein’s first “proxy” intermediate contains the first *k* amino acids of the entire sequence (and the corresponding substructure of the native structure), the second “proxy” intermediate contains the first 2*k* amino acids of the entire sequence (and the corresponding substructure of the native structure), etc.; this continues until arriving at the last “proxy” intermediate, which captures the entire protein’s sequence (and the entire native 3D structure) (Newaz *et al.* 2022). For discussion of the best choice of *k*, see Fig. S6, available as supplementary data at *Bioinformatics* online. The set of all “proxy” intermediates for a protein aims to mimic the process of co-translation. We say “mimic”, because unfortunately this procedure is unable to capture any conformational changes of an intermediate over time, i.e. any actual dynamics of the co-translational folding process, because all of the intermediates are extracted from the same, static native structure. Although these intermediates do not explicitly model co-translational folding as it occurs *in vivo* (hence the term “proxy”), they were the most practical large-scale method for mimicking co-translational folding, i.e. the best one could do on a large scale at the moment without significant computational and methodological innovation.

**Figure 5:**
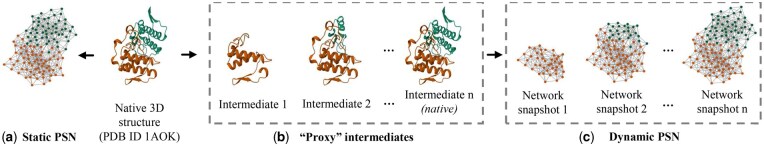
Illustration of (a) a static protein structure network (PSN), (b) “proxy” co-translational intermediates, and (c) dynamic PSN, all for the same protein with PDB ID 1AOK. The static PSN captures the native structure of the protein. “Proxy” co-translational intermediates capture gradually increasing portion of the protein sequence and the corresponding portion of the native 3D structure, with the last “proxy” intermediate capturing the entire sequence and native structure. Each “proxy” intermediate (intermediate 1, 2, …, *n*) corresponds to a network snapshot (network snapshot 1, 2, …, *n*, respectively) in the dynamic PSN. The figure has been adapted from Newaz *et al.* (2022).

We evaluated our approach for extracting “proxy” structural intermediates in the task of protein structure classification (PSC) (Koehl 2006, Newaz *et al.* 2020a), which is closely related to protein function prediction (Gligorijević *et al.* 2021). Traditional 3D-structural features for PSC are extracted directly from 3D structures. Instead, one can first model a 3D structure as a protein structure network (PSN) (Faisal *et al.* 2017). Then, a wealth of network-based methods can be used to extract network features for use in the PSC task (which otherwise could not be extracted directly from 3D protein structures). Our lab had already demonstrated that using PSN features extracted from native structures often proved to be more accurate and typically faster than using sequence and non-network-based 3D structural approaches in the PSC task (Newaz *et al.* 2020a). However, traditional PSN approaches (including our lab’s previous approaches) had modeled a native 3D structure as a *static* PSN (Fig. 5a), because experimental data on dynamics (i.e. on co-translational intermediates) are lacking (Section 2.2). However, with the availability of “proxy” co-translational intermediates, more recently, we were able to model a native 3D structure as a *dynamic* (Fig. 5c) rather than static PSN (Newaz *et al.* 2022); the desire to model a native 3D structure as a dynamic PSN, in order to capture, even implicitly, the dynamics of protein folding, was the reason why we came up with the idea of “proxy” co-translational intermediates. We demonstrated that modeling a 3D structure as a dynamic PSN (Newaz and Milenković 2019, Newaz *et al.* 2022) performed better in the PSC task than modeling it as a static PSN. These results confirmed that considering dynamic information, even only as “proxy” co-translational intermediates derived from native structures, could improve insights into folding-related phenomena.

#### 3.2.3 “Proxy” versus AlphaFold2-predicted co-translational intermediates

The PSC task is not explicitly designed to address protein folding *dynamics*. So, as an original contribution to this current study, we evaluate “proxy” intermediates in a different task more directly related to folding dynamics. Specifically, we assess 3D structural similarity (as measured by TM-score) between experimentally determined intermediates and their corresponding “proxy” intermediates. We do not anticipate a perfect structural match between the two. This is because the former often undergo conformational changes during co-translation (as the TM-scores from Fig. S2, available as supplementary data at *Bioinformatics* online, show), but this is not reflected in the design of the latter (as the “proxy” intermediates are extracted from a native structure). So, the comparison between experimentally determined intermediates and “proxy” intermediates is not our key goal here. Instead, it is to evaluate whether the experimentally determined intermediate conformations are structurally matched better or worse by their corresponding “proxy” intermediates or by their corresponding AlphaFold2-predicted intermediates. If better, this would further highlight AlphaFold2’s limitations in modeling protein folding dynamics.

When we analyze a subset of the four studies/proteins with available experimentally determined 3D structures of co-translational intermediates that meet relevant criteria (Section S3.3, available as supplementary data at *Bioinformatics* online), our findings are as follows (Table 1). The AlphaFold2-predicted intermediates match the experimentally derived intermediates just as poorly as the “proxy” intermediates. All TM-scores are quite low, between 0.21 and 0.53. AlphaFold2 performs better (yet not too well, as indicated by the TM-scores) for a half of the analyzed data, while “proxy” intermediates perform better (also not too well) for the other half. An additional observation is that depending on the protein, AlphaFold2’s predictions for non-native intermediates are of the same fold as proxy intermediates (TM-scores in the 0.59–0.85 range) in some cases, but of different folds than proxy intermediates (TM-scores in the 0.28–0.33 range) in other cases (Table 1).

We showed in our recent study (Newaz *et al.* 2022) that using “proxy” intermediates in a dynamic fashion is a powerful method for capturing more information from native 3D structures in the task of PSC than analyzing the native structures in a traditional, static fashion (Section 3.2.2). In the above analysis, we have shown that “proxy” intermediates perform comparably to AlphaFold2 in the task more closely related to protein folding dynamics—predicting 3D structures of co-translational intermediates. Again, we do not aim to imply that “proxy” intermediates are an appropriate approach for modeling 3D structures of folding intermediates, and thus for capturing folding dynamics. Instead, our findings imply that AlphaFold2 does not consistently outperform the baseline “proxy” intermediates in the second task, and it also most often yields TM-scores below 0.5 that indicate a changed fold, all of which underscores the need for new types of approaches that will be capable of accurately modeling folding dynamics and predicting 3D structures of intermediates.

## 4 Discussion and concluding remarks

Our study (and others) highlights a fundamental barrier to advancing the understanding of protein folding dynamics: the near-total absence of 3D structural data on folding intermediates, in the context of both post-translational and co-translational folding. For post-translational folding, our literature search has revealed only two studies that report experimentally determined 3D structures of post-translational intermediates [we have also identified 30 proteins with less-detailed 3D structural data capturing coarser-grained folding dynamics information on which portion of a protein’s *native* 3D structure folds first versus second/last (Zhao *et al.* 2024a, 2024b)]. For co-translational folding, we have identified only four studies that report experimentally determined 3D structures of co-translational intermediates. Each of these two plus four proteins is constrained by a limited number of intermediates, and most by short length.

While thermodynamic and kinetic datasets do exist on a somewhat broader scale for post-translational folding, they typically provide a single quantity per protein rather than per intermediate along a folding pathway. Even in the best cases, where some proteins have a measurement per intermediate, the data are only quantitative, lacking 3D spatial information. For co-translational folding, the situation is even more sparse; there are no centralized resources containing data of these types; instead, only a few isolated studies provide such data, typically with one protein per study. As a result, while thermodynamic and kinetic data allow for quantitative analyses of post-translational folding, their lack of accompanying 3D structural information constrains the depth of such analyses. For co-translational folding, the near-complete absence of both quantitative and 3D structural data for intermediates leaves little means for any analysis.

Given the lack of experimentally determined 3D structural data on intermediates, it is natural to ask whether computational methods—especially those that have demonstrated success in predicting native 3D structures—can help bridge the gap. Our findings from Section 3.2, along with those of Outeiral *et al.* (2022) discussed in Section 3.1, provide a strong caution against this assumption. These findings reinforce the conclusion that existing structural prediction methods do not generalize well outside of native conformations, and thus cannot be expected to accurately predict 3D structures of intermediates along a folding pathway.

So, how may this be achieved (more) accurately? One possibility may be to retrain existing structure prediction methods with data on folding intermediates, and in doing so, re-engineer these approaches to predict the 3D structures of folding intermediates. However, this direction is limited by the near-total lack of experimentally determined 3D structural data on intermediates, both for post-translational and co-translational folding.

Another possibility may be running AlphaFold2 using advanced input adjustments to generate an ensemble of structural models for full-length sequences (Wallner 2023, Monteiro da Silva *et al.* 2024, Raouraoua *et al.* 2024). While approaches like these can potentially reveal multiple plausible conformations, we hypothesize that they are unlikely to be capable of accurately generating non-native intermediate structures (for either a partially folded or full-length sequence); fundamentally, these models are trained to predict stable, native structures based on multiple sequence alignment information. Moreover, beyond these advanced ways to run AlphaFold2 (or any other existing method for predicting native 3D structures), there also exist specialized AI models explicitly designed to generate diverse protein conformations in the task of protein conformational ensemble prediction (Jing *et al.* 2023, 2024, Wang *et al.* 2024, Lewis *et al.* 2025). However, because unstable non-native intermediate structures can differ significantly from native conformations, we hypothesize that these tools are also likely ill-suited for predicting protein folding pathways. Although we believe both of these hypotheses to be true, definitive verification would require empirical testing; this is outside the scope of the current study and is a subject of future work.

Yet another possibility may be to develop methods that more accurately account for the underlying biochemical and physical factors driving protein folding dynamics, such as the geometry of the ribosome (and its tunnel) or other mechanisms by which the ribosome thermodynamically regulates co-translational folding, presence of chaperones, or vectorial, time-dependent nature of translation (in the case of co-translational folding) (Nilsson *et al.* 2015, Agirrezabala *et al.* 2022, Chan *et al.* 2022, Wang *et al.* 2025). In a bit more detail regarding the importance of the ribosome’s role: it is understood that disruptions to translation by the ribosome—such as artificially induced pauses—can trigger widespread protein misfolding and aggregation, underscoring how tightly coordinated elongation and folding must be. The ribosome helps maintain this coordination by preventing premature co-translational misfolding by the unfolded nascent chain, lowering the entropic penalty of protein folding, and participating with chaperones in assisting folding even before protein synthesis is complete (Duran-Romaña *et al.* 2025). Complementing these biochemical observations, simulation studies that explicitly model the ribosomal exit tunnel and stepwise translation have predicted how the ribosome influences the conformations adopted by nascent peptides at the moment of release—structures that typically differ from their native fold (Tao and Xiao 2025).

In addition to the above biochemical and physical factors driving protein folding dynamics, incorporating codon usage patterns might improve the prediction of co-translational folding pathways because it serves as a proxy for translation rates, which are evolutionarily tuned to regulate the timing of protein synthesis (O’Brien *et al.* 2014). For instance, slower translation rates allow the N-terminal portion of a protein to fold independently before the synthesis of the C-terminal portion is complete, while faster translation rates can help prevent the buildup of certain folding intermediates (Moss *et al.* 2024). Even synonymous codon usage—where different codons code for the same amino acid—can affect translation speed and, as a result, impact co-translational folding (Newaz *et al.* 2020b, Moss *et al.* 2024).

Recent efforts have begun to demonstrate the potential of considering these factors for better prediction of folding pathways. For post-translational folding, Pathfinder (Huang *et al.* 2023), PAthreader (Zhao *et al.* 2023), and FoldPAthreader (Zhao *et al.* 2024b) were introduced (Section 3.1). For co-translational folding, we and others have modeled the vectorial nature of protein synthesis by explicitly capturing increasingly longer subsequences of a protein and then predicting their 3D substructures as the structures of co-translational intermediates; the latter has been achieved in four distinct ways, as follows.

First, we previously introduced “proxy” intermediates (Newaz *et al.* 2022)—3D substructures representing progressively longer C-terminal sequence fragments of a protein extracted *naively from its native structure—*to model co-translational folding; while “proxy” intermediates do not capture protein folding dynamics *explicitly*, we showed that their analysis is nonetheless significantly more accurate than traditional analyses of only the native structures in the task of predicting protein structural classes (Section 3.2.2).

Second, in the current study, we have used a more advanced idea of predicting 3D structures of co-translational intermediates by feeding progressively longer C-terminal sequence fragments of a protein into AlphaFold2; however, this approach was unable to accurately predict the existing experimentally determined co-translational intermediates (Section 3.2.1), likely because it does not effectively learn the kinetics (and thus dynamics) of protein folding (Outeiral *et al.* 2022).

Third, a different existing structure prediction method—which predicts protein folding pathways while accounting for *non-native* interactions and which considers some key principles of co-translational folding—was used on increasingly longer subsequences of a protein to predict their 3D structures. Namely, the study by Wang *et al.* (2025)—which we again note appeared as *after* the completion of all of our analyses—used Monte Carlo simulations that “recapitulate intrinsic properties of co-translational folding, such as the sequential emergence of the peptide and its propensity to form secondary and tertiary structures” and also “provide insights into the thermodynamic stability and dimensions of folding intermediates, which determine whether structural elements can form inside the narrow ribosome exit tunnel” (Wang *et al.* 2025). To evaluate the predicted 3D structures resulting from these simulations, Wang *et al.* (2025) performed *in vitro* experiments using truncated protein domains (sequence fragments of increasing length) attached to a ribosome that mimicked different stages of co-translation. The experimentally measured structural compactness and stability of these domains were then compared to the corresponding simulation-predicted conformations and stability profiles, and the two seemed to align.

Fourth, another simulation-based approach that explicitly models the differences between co-translational folding and post-translational (free) folding is the framework introduced by Tao and Xiao (2025). This study—which also appeared *after* the submission of our article—developed a framework that incorporates both a simplified geometric model of the ribosomal exit tunnel and a residue-by-residue translation process, enabling atomistic molecular dynamics simulation of both co-translational folding and free folding of a protein’s 3D structure. This approach was designed to address two specific questions. One question was what structure the nascent peptide adopts upon expulsion from the ribosomal exit tunnel. Using over 8 ms of simulations across three proteins with distinct topologies, Tao and Xiao (2025) found that co-translational folding influenced the nascent chain to adopt a more helix-rich structure with less long-range contacts upon exit from the ribosome compared to post-translational folding. The other question was how this structure evolves during subsequent folding. By comparing co-translational and post-translational trajectories, subsequent folding pathways were found to be very similar, but with co-translational folding favoring conformations that promote faster folding. Note that Tao and Xiao (2025) did not evaluate/validate their simulated co-translational pathways against experimentally determined 3D intermediate structures; rather, they used their simulated pathways to examine how ribosomal confinement and protein translation bias early conformations and pathway selection. By accounting for these biases, *in vivo* observations that once appeared inconsistent with *in vitro* observations—such as differences in folding efficiency or misfolding propensities—could be understood as natural consequences of ribosome-guided pathway selection.

When a new method becomes available for predicting the 3D structures of folding intermediates, an immediate challenge will be the evaluation of its predictions, i.e. assessment of whether the predicted 3D structural intermediates match biological reality. This can be approached in several ways.

First, ideally, evaluation would involve 3D structural comparison between predicted and experimentally determined folding pathways. Although such experimental data are scarce, they are not entirely absent; such data exist for post-translational as well as co-translational folding for a handful of proteins (Section 2); this is similar to the initial scarcity of available protein 3D structures at the inception of PDB (see below and Fig. S7a, available as supplementary data at *Bioinformatics* online). Given a predicted pathway and an experimentally determined pathway, one strategy to perform their 3D structural comparison could be to generalize established structural similarity measures from comparing two native structures to comparing corresponding pairs of intermediates along the two pathways. This is precisely the strategy we have used to evaluate AlphaFold2-predicted co-translational intermediates in Section 3.2.1. While we have used TM-score (for reasons discussed in Section S2.1, available as supplementary data at *Bioinformatics* online), additional 3D structural similarity measures can also be used, which are sometimes in agreement with and sometimes complementary to each other, depending on whether they are global versus local, superposition-based versus superposition-free, or considering all atoms versus only selected subsets of atoms (Olechnovič *et al.* 2019). Another quite natural strategy for 3D structural comparison of the two pathways could rely on dynamic PSNs. Namely, PSNs have been powerful in tasks such as protein structural classification (Faisal *et al.* 2017, Newaz *et al.* 2020a) as well as protein function prediction (Gligorijević *et al.* 2021). And of all PSN types, dynamic PSNs have shown the greatest accuracy (Newaz *et al.* 2022). Dynamic PSNs were introduced exactly for the purpose of modeling 3D structures of intermediates along a co-translational pathway (Section 3.2.2). So, each of a predicted pathway and an experimentally determined pathway can naturally be converted into its respective dynamic PSN. Then, the two pathways, i.e. their dynamic PSNs, can be compared using a wealth of approaches for dynamic network comparison (Vijayan *et al.* 2017, Zitnik *et al.* 2024). This is especially true given the rapid advancement of artificial intelligence, including deep learning on graphs (Gligorijević *et al.* 2021, Zitnik *et al.* 2024), as well as geometric deep learning combined with network analyses (Morehead and Cheng 2024a,b); these kinds of approaches have already shown strong performance in a range of structural biology tasks such as drug binding, protein–protein interaction prediction, and fold classification (Zitnik *et al.* 2024).

Second, when experimentally determined 3D structures of intermediates along a pathway are unavailable, one evaluation strategy could be to use quantitative data (e.g. folding rate constants or thermodynamic stability), which are more widely available than 3D structural data, especially for post-translational intermediates, like what Outeiral *et al.* (2022), Huang *et al.* (2023), and Zhao *et al.* (2023, 2024b) did (Section 3.1). Third, evaluation could involve performing in-house wet-lab experimental validation, as done by Wang *et al.* (2025), per our discussion earlier in this section.

In summary, the problem of protein folding dynamics is challenging, including due to data sparsity. However, such type of challenge is not without precedent. At the inception of PDB (wwPDBconsortium 2019) in 1976, only 13 structures were available. For its first decade, structural growth was slow, with only 6–32 additional structures being added to PDB each year (Fig. S7a, available as supplementary data at *Bioinformatics* online). Yet over time, PDB has grown to include over 200 000 structures (Fig. S7a, available as supplementary data at *Bioinformatics* online). In parallel, the launch of the Critical Assessment of protein Structure Prediction (CASP) competition (Yuan *et al.* 2026) in 1994, with 27 teams and 186 predictive methods (Fig. S7b, available as supplementary data at *Bioinformatics* online), sparked rapid progress in native structure prediction, culminating in tools like AlphaFold2 and an explosion of predicted 3D structures—with over 214 million in the AlphaFold Protein Structure Database alone (Varadi *et al.* 2024). This history suggests that major progress can begin with limited data. Such a growing trajectory, likely with a much faster timeline due to developments in artificial intelligence and machine learning, may be possible for data on 3D structures of folding intermediates if the community begins to systematically organize existing and generate new 3D structural data, however sparse; this has been exactly a key goal of our article. Like the early PDB and CASP efforts on native structures, such a foundation could catalyze innovation in intermediate-specific experimental technologies as well as computational prediction methods (importantly, including evaluation frameworks). A valuable and recent experimental attempt toward detecting co-translational intermediates is a modification to existing NMR spectroscopy, called ${\;}^{19}F$ NMR, which can detect and measure stable structural states accessed by a protein during co-translation (Chan *et al.* 2022).

Addressing the current impasse will require coordinated, closely intertwined efforts across wet-lab experimental and computational communities. On the experimental side, new technologies are needed to capture transient 3D structures of intermediates with higher resolution and faster acquisition times. On the computational side, more efforts are needed to develop intermediate-specific prediction methods informed by curated experimental 3D structural data as well as kinetics or thermodynamics data. Additionally, we encourage the computational community to include analyses of computational complexity as well as results on running times and memory usage when evaluating new methods, as such key data are often overlooked in the current literature. Developing accurate and efficient computational approaches is only part of the endeavor; ensuring these methods are user-friendly and broadly accessible is equally critical for their widespread adoption. This includes offering intuitive interfaces and well-documented software, providing open-source implementations, and ensuring compatibility with platforms and workflows commonly used by the community.

## Supplementary Material

btag020_Supplementary_Data

## Data Availability

Only existing, already published data and software are used in this article, all of which are publicly available and appropriately referenced throughout the article. For easiness and reproducibility, we provide a consolidated list of these, along with our predicted **3D** structural data on “proxy” and AlphaFold2-predicted co-translational **p**rotein **f**olding **i**ntermediates, at https://github.com/Aywells/3Dpfi or https://academicweb.nd.edu/∼cone/3Dpfi/.
