# Supplementary material for: Unavailability of experimental 3D structural data on protein folding dynamics and necessity for a new generation of structure prediction methods in this context
Source: Bioinformatics. 2026 Jan 20;42(2):btag020. doi: 10.1093/bioinformatics/btag020 (PMC12926781; doi:10.1093/bioinformatics/btag020)
Supplement: btag020_Supplementary_Data [file btag020_supplementary_data.pdf]

Supplementary information for: Unavailability of experimental 3D structural data on protein folding dynamics and necessity for a new generation of structure prediction methods in this context

Aydin Wells[1], Khalique Newaz[2], Jennifer Morones[1], Jianlin Cheng[3], Tijana Milenković[1]

[1]Department of Computer Science and Engineering, University of Notre Dame, USA
[2]Institute for Computational Systems Biology, University of Hamburg, Germany
[3]Department of Electrical Engineering and Computer Science, University of Missouri, Columbia, USA

S1 Supplementary data

S1.1 Additional details on kinetics and thermodynamics data related to co-translational folding

Here, we complement Section 1 in the main paper by discussing existing kinetics and thermodynamics data related to co-translational folding.

For co-translational folding, we could not identify any organized database containing kinetics or thermodynamics data. Instead, we could identify some isolated studies that provide data of these types for a handful of proteins, as follows. Samelson et al. (2018) reported kinetics (co-translational folding rate) data for a protein called HaloTag, Farías-Rico et al. (2018) studied the thermodynamic stability of the S6 protein, and Kelkar et al. (2012) captured kinetics data for intermediates of the protein mCherry at specific time points.

S1.2 Additional information about the studies on experimentally determined 3D structures of post-translational intermediates

Here we complement Section 2.1 in the main paper by providing more details about the two studies on post-translational intermediates:

Neudecker et al. (2012) addressed the question of how folding intermediates contribute to amyloid fibril formation, a key process in many neurodegenerative disorders. While such intermediates have been implicated in aggregation, the structural mechanisms underlying this transition remain poorly understood. To investigate this, Neudecker et al. (2012) used NMR spectroscopy to determine the structure of a post-translational intermediate of the Fyn SH3 domain. Neudecker et al. (2012) found that the intermediate exhibited a disordered C-terminus, exposing an aggregation-prone β-strand. Two pathway intermediates are provided in the study: the first intermediate is an "early-stage" structure (PDB ID: 2L2P) and the second intermediate is the native structure (PDB ID: 2L25) of the SH3 domain.

Zhou et al. (2008) explored the folding mechanisms of ribonuclease H (RNase H) in order to enhance understanding of the principles governing protein folding dynamics. Utilizing multidimensional NMR, Zhou et al. (2008) identified a compact intermediate of RNase H that formed rapidly during folding, exhibiting a native-like core of helices. The study's findings supported a hierarchical folding mechanism, where secondary structures formed first, followed by tertiary contacts. Additionally, Zhou et al. (2008) findings indicated that the intermediate was well-defined and folded into its native structure more quickly because of this intermediate. Two post-translational intermediates are provided in the study: the first intermediate is an "early-stage" structure (PDB ID: 2RPI) and the second intermediate is the native structure (PDB ID: 1RIL) of the RNase H protein.

S1.3 Additional information about the studies on experimentally determined 3D structures of co-translational intermediates

Here we complement Section 2.2 in the main paper by providing more details about the four studies on co-translational intermediates:

Agirrezabala et al. (2022) investigated how early folding events are influenced by the ribosome – and hence the shape of protein structures – during translation. Specifically, Agirrezabala et al. (2022) observed the folding of a β-barrel protein (specifically a cold shock protein (CspA)) and reported its interactions with the ribosome during translation. Using cryo-EM, Agirrezabala et al. (2022) captured a co-translational intermediate of CspA that revealed an initial α-helical conformation that formed within the ribosome's exit tunnel before transitioning into its native β-strand structure upon emergence. The study's findings emphasized the ribosome's role in shaping early folding events and suggested that co-translational folding may be a common feature of β-barrel proteins. Agirrezabala et al. (2022) provided the native structure of the CspA protein (PDB ID: 1MJC), three different conformations of the CspA protein when 27 amino acids have already been translated (PDB ID: 7NWW, 7OIF, 7OIG), and two different conformations of the CspA protein when 70 amino acids have already been translated (PDB ID: 7OT5, 7OII).

Hanazono et al. (2018) explored the co-translational folding of nascent polypeptide chains, specifically focusing on the λ repressor N-terminal domain, a small α-helical protein, to uncover the atomic-level details of this process. Using circular dichroism (CD) spectroscopy, Hanazono et al. (2018) examined intermediate-length variants of the λ repressor to capture structural intermediates during co-translational folding. The study found that partial helices formed within the ribosome's exit tunnel, with increasing chain length leading to progressive stabilization of secondary and tertiary structure. This suggested a stepwise folding process where local α-helical segments formed early and guided subsequent structural changes. Their results highlighted how the ribosome constrains and influences protein stability and function. Hanazono et al. (2018) reported the native structure of the λ repressor (PDB ID: 5ZCA), and the structures of two different co-translational folding intermediates; the first of the two captures the structure of the λ repressor when 20 amino acids have already been translated (PDB ID: 3WOA) and the second captures the structure of the λ repressor when 45 amino acids have already been translated (PDB ID: 1LMB).

Hanazono et al. (2016) investigated the co-translational folding of nascent proteins, which are affected by the rate of translation by the ribosome. While fully translated proteins are capable of achieving their native conformation, nascent protein structures primarily begin folding co-translationally at their N-terminal regions; however, the transient structures of intermediates involved in this process remain poorly understood. Hanazono et al. (2016) focused on the early folding events of the WW domain, a small β-sheet protein, during translation. Using CD spectroscopy, N-terminal fragments of the WW domain were examined to determine how partial sequences adopted structure before arriving at the native structure. Hanazono et al. (2016) showed that isolated N-terminal fragments lack stable β-sheet formation, suggesting that the WW domain required a sufficiently long polypeptide chain before β-strands can properly fold. This contrasts with α-helical structures, where partial helices formed early. Hanazono et al. (2016) findings highlighted a cooperative folding mechanism for β-sheet proteins, where both chain length and long-range molecular interactions played a key role in stabilizing the WW domain's structure. The study reported the native structure of the WW domain (PDB ID: 5B3Z), and the structures of three different N-terminal fragments of the WW domain: the first of the three captures the structure of the WW domain when 11 amino acids have already been translated (PDB ID: 3WOA), the second of the three captures the structure of the WW domain when 17 amino acids have already been translated (PDB ID: 5BMY), and the last of the three captures the structure of the WW domain when 19 amino acids have already been translated (PDB ID: 5B3Y).

Cabrita et al. (2016), using NMR spectroscopy, analyzed folding of FLN5 in isolation (i.e., its post-translational folding) vs. folding of FLN5 in the presence of the ribosome (i.e., FLN5's co-translational folding). Specifically:

- Regarding the former: isolated FLN5 was observed to fold spontaneously, corresponding to FLN5's native 3D structure; we show this structure, reported by Cabrita et al. (2016), as intermediate 1 in Fig. 3 of the main paper (PDB ID: 1QFH – now updated to 6G4A).
- Regarding the latter: Cabrita et al. (2016) examined how much time needed to pass between the entire FLN5 being out of the ribosome tunnel before obtaining its native structure (i.e., a structure closely

matching the native structure of isolated FLN5). To be able to measure this, Cabrita et al. (2016) attached to FLN5 an increasingly longer portion of the sequence of FLN6, with FLN5 being at the N-terminal of the combined sequence, and FLN6 being at its C-terminal. Then, appending a shorter subsequence of FLN6 to (the full-sequence of) FLN5 meant less time had passed since FLN5 was translated by the ribosome, while appending a longer subsequence of FLN6 to (the full-sequence of) FLN5 meant more time had passed since FLN5 was translated by the ribosome. What Cabrita et al. (2016) then found was that FLN5 could obtain the native structure in the presence of the ribosome only when a longer (full) subsequence of FLN6 was appended to it. In other words, the complete sequence of FLN5 had to emerge well beyond the ribosome tunnel before it could acquire its native structure (while we note again that FLN5 in isolation folded spontaneously). Cabrita et al. (2016) concluded this to be evidence that the ribosome modulates the co-translational folding process of FLN5. Cabrita et al. (2016) reported three distinct 3D structural confirmations of the combined FLN5+FLN6 sequence, corresponding to intermediate 2 – conformations a, b, and c – in Fig. 3 in the main paper (PDB ID: 2N62). Note that per the above discussion of Cabrita et al. (2016) results, (only) the FLN5 portion of these three 3D structural confirmations of FLN5+FLN6 (i.e., the green part of confirmations 2a, 2b, and 2c in the "Cabrita et al., (2016)" portion of Fig. 3 in the main paper) is a (close) structural match to the isolated native 3D structure of FLN5 (i.e., intermediate 1 in the "Cabrita et al., (2016)" portion of Fig. 3 in the main paper).

S2 Supplementary methods

S2.1 Additional details about our use of TM-score

Here we provide details on our use of TM-score (Xu and Zhang, 2010, Zhang and Skolnick, 2004).

TM-score is a widely used quantitative measure for assessing the level of similarity between two 3D structures. It is a *global* measure, meaning that it evaluates the overall agreement of entire folds between two 3D structures, even if some of their peripheral regions might differ (Zhang and Skolnick, 2004).

In contrast to global 3D structural similarity measures, *local* measures assess structural similarity at a finer spatial scale; these measures typically involve calculating the spatial distance deviations of pairs of residues (typically $C\alpha$ atoms) within a *local neighborhood*, and then aggregating scores over only short-range residue interactions. As such, local structural similarity measures are sensitive to local distortions, making them useful for evaluating the accuracy of smaller structural regions regardless of the global fold (Mariani et al., 2013).

It was recently shown that global measures of 3D structural similarity – including TM-score and Global Distance Test (GDT) – are "extremely highly correlated" (Olechnovič et al., 2019), which supports the use of TM-score as a representative global measure. The same study found that local measures – including local Distance Difference Test (lDDT), Recall, Precision and F-measure (RPF), and Contact Area Difference (CAD) – are also "highly correlated" with each other (Olechnovič et al., 2019). Further, that study found that the local similarity scores are often in agreement with global ones, as well as that the methods for predicting 3D structures (referred to as "models" in that study) that are selected as the best with respect to local measures (including lDDT and RFP) are typically also among the best methods selected according to the global measures (including TM-score and GDT) (Olechnovič et al., 2019). Yet, the two groups of measures, global vs. local, as well as individual measures within each group, often have (dis)advantages (Olechnovič et al., 2019).

Since one aim of our study is to determine whether two compared 3D structures (as described in cases (i) and (ii) below) have the same *overall* fold, we use TM-score, which is global, as our primary measurement for assessing 3D structural similarity. As mentioned above, local structural similarity measures might provide valuable complementary information in certain aspects, particularly when the global fold is preserved. However, we argue that if the global fold is not preserved between the compared 3D structures – as we have observed in our analyses of non-native folding intermediates, where TM-scores are almost always below 0.5 – identifying specific regions of local deviation would offer limited structural insights, as the underlying folds

are fundamentally distinct. Given this, and given the above discussion of frequent agreement/consistency as well (rather than just complementarity) between results of global and local measures, we believe that the use of TM-score as our measure of choice in our study is justified as well as sufficient.

We use the TM-score (Zhang and Skolnick, 2004) software available through the Zhang lab's web server (https://zhanggroup.org/TM-score/). We compare 3D structures of two intermediates (i.e., their sequences) in terms of TM-score as follows. We either compare (i) the experimentally determined 3D structure of a given sequence to the experimentally determined 3D structure of the same sequence *at a different time* (Section 2 in the main paper), or (ii) the *experimentally determined vs. predicted* 3D structures a given sequence (Section 3.2 in the main paper). Because there exist some (minor) discrepancies between the deposited and modeled sequences of a given intermediate (the hatched boxes in Fig. 3 in the main paper) for several of the 15 considered conformation of the 10 considered intermediates (all but the two red conformations of intermediates in Fig. 3 in the main paper), there also may exist discrepancies between the modeled sequences of two compared intermediates. If so, we handle these discrepancies when computing TM-scores between two modeled sequences as follows. For case (i) above, we first identify and extract the longest common contiguous subsequence (LCCS) shared between the experimentally determined compared modeled sequences. Each of the two resulting LCCS modeled sequences is then converted into PDB format and uploaded to the TM-score web server mentioned above using the "Structure 1" and "Structure 2" input fields. Then, the web server returns the corresponding TM-score. For case (ii) above, recall that we predict a 3D structure for an intermediate by inputting the *deposited* sequence of the intermediate into AlphaFold2 (Section 3.2.1 in the main paper). Then, when we compare a predicted 3D structure and its experimentally determined counterpart. Here, we first extract the LCCS between the deposited sequence of the predicted 3D structure and the modeled sequence of its corresponding experimentally determined 3D structure. Second, the two resulting 3D structures corresponding to the two LCCSs are then converted into PDB format and uploaded to the TM-score web server mentioned above using the "Structure 1" and "Structure 2" input fields. Then, the web server returns the corresponding TM-score. Note that in either of cases (i) and (ii) above, we do not need to normalize a given TM-score with respect to the length of either of the two input 3D structures, because we compare only the LCCS shared between the pair; i.e., the two structures inputted into the TM-score web server always have the exact same sequence.

To reemphasize, this methodology is applied uniformly across all analyses presented in Sections 2 and 3.2 of the main paper where TM-score is employed.

TM-scores range from 0 to 1, with higher values indicating greater structural similarity between protein 3D structures. In more detail (Xu and Zhang, 2010): A TM-score below 0.17 indicates a random-like structural similarity, i.e. that the probability of finding a TM-score this low from randomly chosen structural pairs is close to 1. A TM-score higher than 0.3 indicates a significantly high structural similarity, i.e. that the probability of finding such a score from random structural pairs is very low. A TM-score of 0.5 is generally considered a key threshold, as a TM-score higher than 0.5 suggests that the compared 3D structures have the same overall fold or similar topology according to structural classifications such as CATH (Greene et al., 2007) and SCOPe (Fox et al., 2014).

S2.2 Additional details on the evaluation strategies used in the Outeiral et al. (2022) study

Here we complement Section 3.2 in the main paper with more details on the evaluation from the study by Outeiral et al. (2022).

Recall that Outeiral et al. (2022) investigated whether 3D structures of *post-translational* intermediates could be accurately predicted with existing methods designed for predicting native 3D structures. They asked this question for eight such methods: AlphaFold2 (Jumper et al., 2021), RoseTTAFold (Baek et al., 2021), trRosetta (Yang et al., 2020), RaptorX (Peng and Xu, 2011), DMPfold (Greener et al., 2019), EVfold (Marks et al., 2011), SAINT2 (de Oliveira et al., 2018), and Rosetta (Schaap et al., 2001). More specifically, for each of these methods, Outeiral et al. (2022) asked whether a given method could predict post-translational pathways that are (i) predictive of a protein's folding kinetics class (e.g. two-state or multi-state), and (ii)

correlate with experimentally measured folding rate constants. More details about these two evaluation tasks are as follows.

For the first evaluation task, the methods' predicted pathways were used to classify whether the given protein folds through two-state or multi-state kinetics, in both an unsupervised and supervised manner. Multiple measures of method performance accuracy were used, including the accuracy, F1-score, and area under the receiver-operating characteristic (AUROC) curve. In the text, we summarize the results with respect to AUROC as a representative measure for illustration purposes (where AUROC score of 0.50 indicates random performance, and the higher AUROC score, the better the given method). The AUROC results (for 10 pathways per method per protein, except for AlphaFold2 with one pathway per protein) are as follows. All methods achieved statistically significant yet quite modest performance, with AUROC scores between 0.56 and 0.675. The actual protein structure prediction methods were compared to a trivial baseline, namely a simple linear classifier based solely on chain length. Surprisingly, this simple baseline outperformed all structure prediction methods, with AUROC score of 0.739. Results were qualitatively similar for the other performance accuracy measures and when predicting 200 pathways per method per protein. Full results for this evaluation task are shown in Supplementary Table S1. A key conclusion from this analysis was that this sequence-agnostic baseline surpassed all structure-based methods in predicting folding kinetics, indicating that the predictive signal captured by current structure prediction tools is weak.

For the second evaluation, the methods were evaluated based on how well their predicted pathways could capture the folding rate constants of proteins that undergo two-state folding kinetics. To test this, Outeiral et al. (2022) selected the 79 proteins that had experimentally determined folding rate constants and had at least one pathway classified as two-state. Within these, only the predicted pathways classified as two-state were retained. For each retained pathway, Outeiral et al. (2022) determined the relative frame in which folding occurred, defined as the point of maximal increase in native contacts. Outeiral et al. (2022) then examined whether this position along the pathway correlated with the experimentally measured folding rate constants. Outeiral et al. (2022) compared these correlations of the structure prediction methods to correlations obtained using two baseline methods – average contact order and protein chain length – both being known predictors of folding rates. The results showed that chain length had the strongest (and correct-sign) correlation, outperforming contact order and any structure prediction method. Most structure prediction methods showed weak or insignificant correlations, and in some cases even the wrong sign. Only AlphaFold2 and RoseTTAFold showed modest, correctly signed correlations, hinting at a limited signal. Outeiral et al. (2022) findings suggested that while post-translational folding pathways predicted by structure prediction methods may resemble real post-translational pathways in some aspects, they fail to meaningfully correlate with experimental folding rate data. The original results for the second evaluation task by Outeiral et al. (2022) can be found in Supplementary Fig. S3.

S3 Supplementary results

S3.1 Results on structural similarities between conformations of intermediates for the same protein, for experimental co-translational folding data

Here we complement Section 2.2 in the main paper by reporting key observations and methodological details about structural similarities in terms of TM-scores between all possible pairs of (partial as well as full) conformations of intermediates of the same protein, for experimental co-translational folding data. Relevant results are shown in Supplementary Fig. S2.

First, we focus on different conformations for the same intermediate, which only the studies by Agirrezabala et al. (2022) and Cabrita et al. (2016) have. In other words, we focus on 1a vs. 1b vs. 1c as well as 2a vs. 2c in the former, and 2a vs. 2b vs. 2c in the latter. All of the corresponding TM-scores are below 0.5, indicating that no two conformations of the same intermediate have the same fold.

Second, we focus on the conformational change of a sequence of an intermediate gradually over time, which all four studies have. For example, we focus on the conformation of a green sequence in one intermediate vs. the conformation of the same green sequence in the next intermediate (for the same protein), or on the

conformation of a green+orange sequence in one intermediate vs. the conformation of the same green+orange sequence in the next intermediate (for the same protein). Overall, we observe quite a large conformational change of the same sequence over time in the presence of additional amino acids being translated and added to the 3D structure, most often resulting in a changed fold of the given sequence during translation. In more detail, for the protein from Agirrezabala et al. (2022), we observe the fold change in all comparisons (all TM-scores are 0.33 or lower). For the protein from Hanazono et al. (2018), we also observe the fold change in all comparisons (all TM-scores are 0.43 or lower). For the protein from Hanazono et al. (2016), three of the four comparisons indicate a fold change (TM-scores of 0.46 or lower), and in the remaining case, there is still a large structural change although not necessarily a fold change (TM-score of 0.58). Finally, for the protein from Cabrita et al. (2016), we see the highest TM-scores, meaning the least amount of conformational change, with TM-scores between 0.77 and 0.82. The higher TM-scores for the Cabrita et al. (2016) protein compared to the other three proteins/studies are expected given the unique nature of the Cabrita et al. (2016) study, as described in the last paragraph of Supplementary Section S1.3, i.e., because intermediate 1 is a native structure of isolated (post-translationally folded) FLN5, and (only) the green portions of intermediates 2a, 2b, and 2c are native structures of co-translationally folded FLN5.

Third, in order to evaluate the effect of time passed, we compare conformations corresponding to the same sequence of an intermediate at closer vs. more distant times. Specifically, for the only two studies that allow for this comparison (because they have more than two distinct intermediates in Supplementary Fig. S2) – Hanazono et al. (2018) and Hanazono et al. (2016) – we compare conformational change of the green sequence from time 1 to time 2, and from time 2 to time 3, against its change from time 1 to time 3. For the protein from Hanazono et al. (2018), we find the following. The green sequence in the first intermediate vs. the green sequence in the second intermediate, as well as the green sequence in the second intermediate vs. the green sequence in the third intermediate (i.e. the two pairs of time-closest intermediates), show a high level of conformational changes (TM-scores of 0.43 and 0.42, respectively). Yet, the green sequence in the first intermediate vs. the green sequence in the third intermediate (i.e. the pair of most time-distant intermediates) show an even greater level of conformational change (TM-score of 0.22). Similarly, in Hanazono et al. (2016), the green sequence in the first intermediate vs. the green sequence in the second intermediate, as well as the green sequence in the second intermediate vs. the green sequence in the third intermediate, show some conformational changes (TM-scores of 0.45 and 0.58, respectively). The green sequence in the first intermediate vs. the green sequence in the third intermediate show an even larger conformational change (TM-score of 0.43).

S3.2 Per-study results of AlphaFold2 predictions of co-translational intermediates

Here we complement Section 3.2.1 in the main paper with *per-study* results related to our analysis of AlphaFold2-predicted co-translational intermediates. Relevant results are shown in Fig. 4 in the main paper.

For the Hanazono et al. (2018) and Hanazono et al. (2016) studies, a common trend in TM-scores is apparent; in each of these studies, AlphaFold2's predicted structure of the last intermediate is highly structurally similar (with TM-scores greater than 0.80) to the last modeled sequence (i.e. intermediate 3 in Hanazono et al. (2018), and intermediate 4 in Hanazono et al. (2016)). This is because the modeled sequence of the last intermediate in each of these studies is the native structure (as reported in it's respective study). However, most of AlphaFold2's predicted structures of earlier intermediates do not have the same fold as their experimental counterparts (with TM-scores lower than 0.50, with the exception of intermediate 2 in Hanazono et al. (2018) with TM-score = 0.53).

For the Agirrezabala et al. (2022) study a similar trend is also observed, but, with different insights between different conformations of intermediates; for conformation 2c, AlphaFold2's predicted structure is highly structurally similar to it (with TM-score = 0.95), because the modeled sequence of this conformation is the native structure. In contrast, for conformation 2a – an experimentally observed non-native structure – AlphaFold2's predicted structure is highly dissimilar to it, close to random (with TM-score = 0.22). Although these two conformations have the same deposited sequences, when given a modeled sequence as

input, AlphaFold2's predicted structure is biased towards predicting a native structure with high confidence, rather than a structure of an intermediate. Furthermore, Alphafold2 cannot correctly predict any of the first intermediate conformations (i.e. conformations 1a, 1b, and 1c, with TM-scores less than or equal to 0.17).

At first glance, for the Cabrita et al. (2016) study, results show a different pattern, with AlphaFold2's predicted structure of the first intermediate being highly structurally similar to the modeled sequence of the first intermediate (TM-score = 0.82), and AlphaFold2's predicted structures of the conformations of the second intermediate not having the same fold for most of the conformations (with TM-scores ranging between 0.45 and 0.53). However, the first intermediate is the native structure (as reported in the Cabrita et al. (2016) study), and the conformations of the second intermediate are non-native structures (even though their green portions in Fig. 3 in the main paper are native structures, per our discussion in the last paragraph of Supplementary Section S1.3). While AlphaFold2 accurately predicts the native structure (i.e. intermediate 1), it is unable to predict any conformations of the second intermediate with high structural similarity to experimental data.

S3.3 Additional details on structural similarity between experimentally determined co-translational intermediates and their corresponding "proxy" vs. AlphaFold2-predicted co-translational intermediates

Here we complement Section 3.2.2 in the main paper with more details about the analysis of 3D structural similarity between experimentally determined intermediates and their corresponding "proxy" vs. AlphaFold2-predicted intermediates.

In this analysis, of the four considered studies/proteins, we focus on those studies that meet two key criteria. First, for a given protein, its native structure must correspond to the intermediate with the longest sequence out of all intermediates. This ensures that the native structure corresponds to the full-length conformation, which is necessary for generating "proxy" intermediates by extracting substructures from the native fold. The protein from the Cabrita et al. (2016) study is excluded from further analysis because it violates this criterion: none of the three conformations of the intermediate with the longest sequence (conformations a, b, and c of intermediate 2) are the native structure; the native structure corresponds to the shortest-sequence intermediate (intermediate 1); for more details, see the last paragraph of Supplementary Section S1.3. Second, for a given protein, only a single conformation should be available for any intermediate. This constraint is desirable because if multiple different conformations exist for the same sequence, AlphaFold2 would incorrectly predict the same 3D structure for all of those confirmations, given their shared sequence. The protein from the Agirrezabala et al. (2022) study fails to meet this criterion, as it has three conformations for its intermediate 1 and two conformations for its intermediate 2; also, the protein from the Cabrita et al. (2016) study fails this criterion too, as it has three confirmations for its intermediate 2. Only the proteins from the Hanazono et al. (2018) and Hanazono et al. (2016) studies satisfy both conditions and are thus considered in the rest of this analysis.

We infer AlphaFold2-predicted intermediates for the proteins in the two considered studies as follows: For the protein in the Hanazono et al. (2018) study, we input the experimentally determined sequence of intermediate 1 into AlphaFold2 to obtain the corresponding AlphaFold2-predicted intermediate; similarly, we input the experimentally determined sequence of intermediate 2 into AlphaFold2 to obtain the corresponding AlphaFold2-predicted intermediate. For the protein in the Hanazono et al. (2016) study, we follow the same procedure using the experimentally determined sequences of intermediates 1 and 2 as inputs into AlphaFold2 to obtain their corresponding AlphaFold2-predicted intermediates. Note that we do not generate AlphaFold2-predicted intermediates for the native structures themselves for the same reason as stated above. In total, two AlphaFold2-predicted intermediates are generated for each protein from each of the two considered studies, and their structural similarities to their respective experimentally determined counterparts are computed using TM-score (these scores were originally computed in Fig. 4 in the main paper, but are shown again for the purpose of this analysis in Table 1 in the main paper).

S4 Supporting figures and tables for the main paper

Study	Protein name	PDB ID	Intermediate	Deposited sequence range (starting - ending)	Modeled sequence range (starting - ending)	TM-score
**Neudecker et al. (2012)**	Fyn SH3	2L2P	***1*** 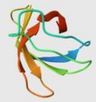	(1 - 66) (early stage)	**(11 - 66)** (early stage)	0.80
		2LP5	***2*** 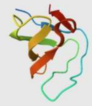	(1 - 66) (final stage)	**(7 - 66)** (final stage)	
**Zhou et al. (2008)**	Ribonuclease H	2RPI	***1*** 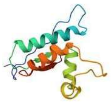	(1 - 112) (early stage)	**(7 - 112)** (early stage)	0.77
		1RIL	***2*** 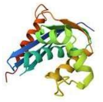	(1 - 166) (final stage)	**(20 - 166)** (final stage)	

Figure S1: Details of the two studies we have identified that report 3D structural data of post-translational intermediates. For each study, i.e. its considered protein, two PDB IDs and two visualizations are shown for the protein's two intermediates (1: "early-stage" or pre-native state; 2: "final stage" or native state). In the fourth and fifth columns, the deposited sequence range and the modeled sequence range for each intermediate is provided, as reported in its respective study and PDB. Note that a bolded model sequence range value indicates that there is a range discrepancy between the reported deposited sequence and the reported model sequence. In the last column, we measure and show the structural similarity between the modeled sequence of the first intermediate and the modeled sequence of the second intermediate using TM-score (Zhang and Skolnick, 2004).

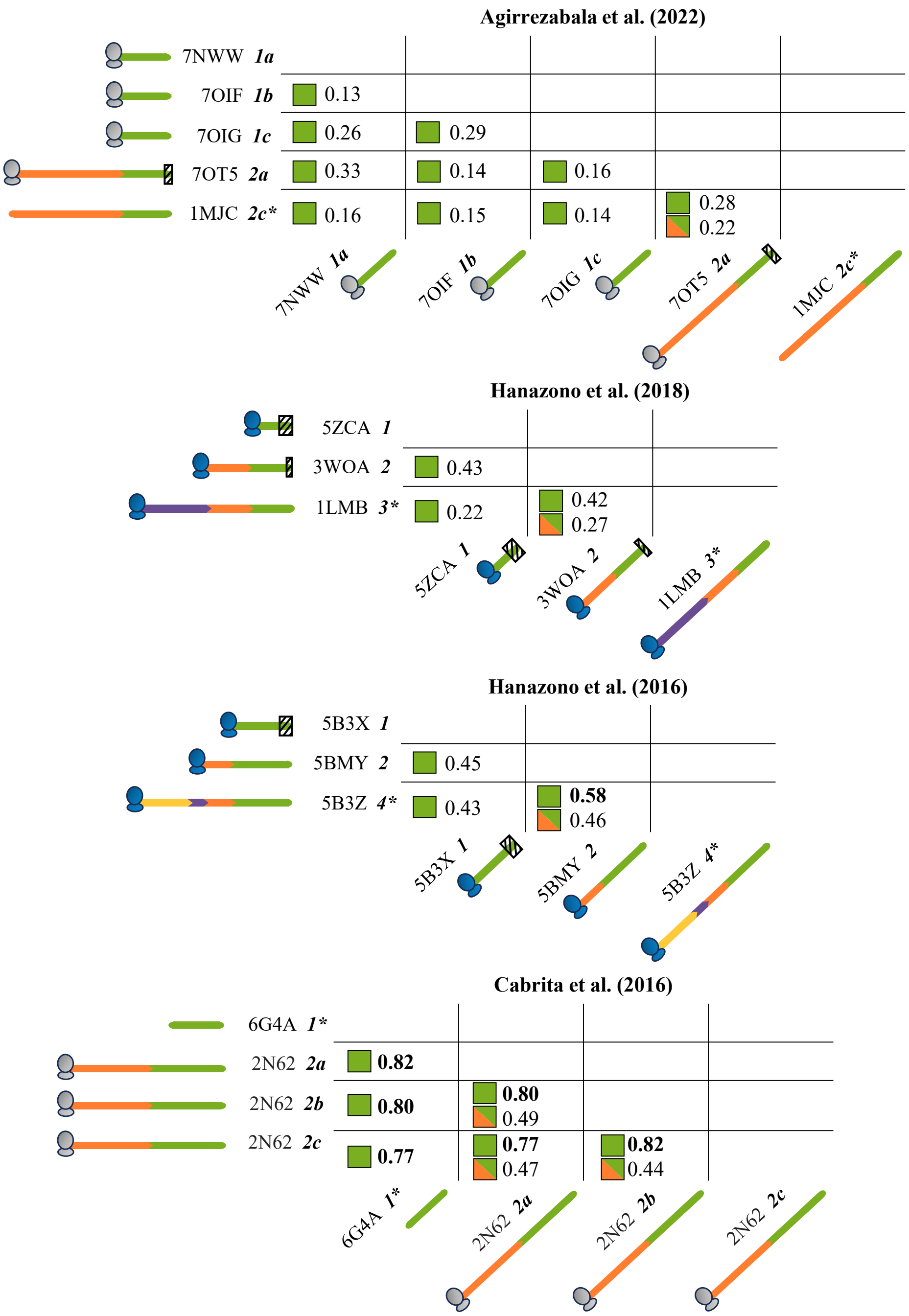

Figure S2: Pairwise structural similarities in terms of TM-score between different combinations of (the conformations of) the co-translational intermediates from the same protein/study. The four proteins/studies considered are the same as in Fig. 3 in the main paper. Their conformations of the intermediates are shown again in the matrix rows/columns of this figure for easiness, with the exception of the conformations with PDB IDs 70II and 5B3Y, which are excluded from any analyses, per the discussion in Fig. 3 in the main paper; all colors and hashed regions in the current figure match those in Fig. 3 in the main paper. There are four matrices of pairwise similarities corresponding to the four proteins/studies, one matrix per protein. In each matrix, TM-scores are shown between all possible pairs of conformations of the intermediates when accounting for their modeled sequence regions. A green box next to a score indicates that only the green regions of the pair of modeled sequences are compared; an orange and green box next to a score indicates that the orange and green regions combined of the pair of modeled sequences are compared. All TM-scores with values higher than 0.50 are bolded, corresponding to structures that have the same overall fold (Zhang and Skolnick, 2004). Only the bottom triangle of the matrix is filled with TM-scores, because the matrix is symmetric.

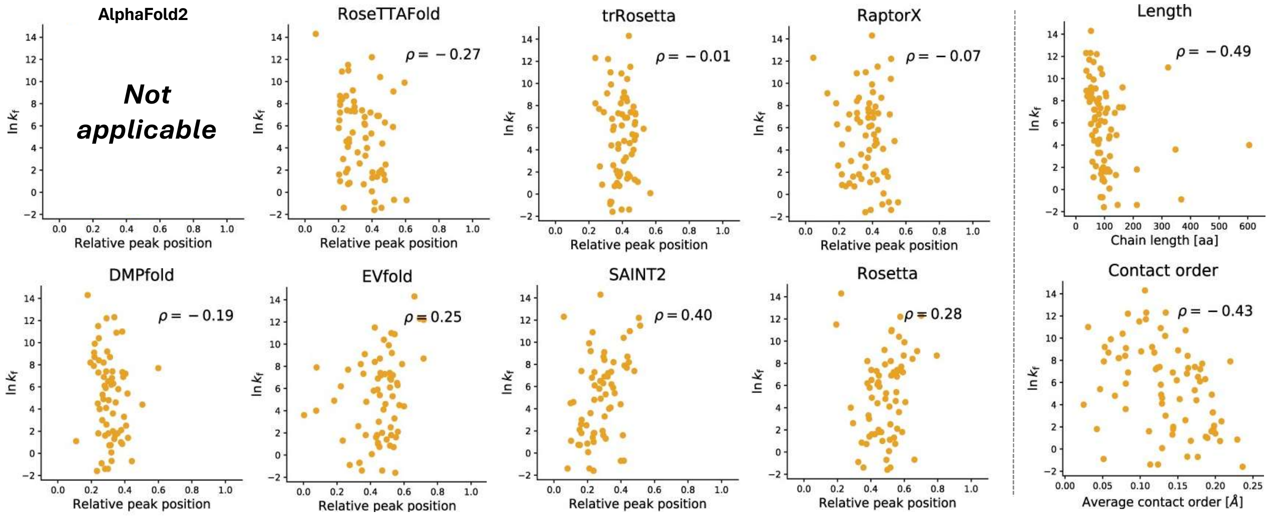

Figure S3: The figure and results from the Outeiral et al. (2022) study on the performance of their eight considered protein structure prediction methods and the chain length ("Length") method as a basic benchmark approach, <u>in their second considered task</u> of examining whether the predicted post-translational pathways correlate with experimentally measured folding rate constants. As stated in the original publication (Outeiral et al., 2022): The illustrated analysis was used to compute the Spearman correlation coefficient ρ between the folding rate constant and folding events in simulated trajectories (i.e., predicted pathways) of the seven considered structure prediction methods, as well as the length of the protein chain and the average contact order of the native structure. Every point represents the average over the maximum number of decoys possible (200 decoys for RoseTTAFold, trRosetta, RaptorX, DMPfold and EVfold; and 10 decoys for SAINT2 and Rosetta). The panel for AlphaFold2 was not included by Outeiral et al. (2022) into this figure, because for AlphaFold2 only one decoy was available; however Outeiral et al. (2022) reported for AlphaFold2 that the correlation coefficient between the relative position of the folding event and the logarithm of the k_f was -0.23", which is of the same order and same sign as RoseTTAFold. In the figure, the best-performing method is Length, as it has the highest absolute-value *and* correctly signed (negative) correlation coefficient of -0.49.

Agirrezabala et al. (2022)

	PDB ID	Intermediate	Modeled sequence range (starting - ending)	TM-score *Rank 1*	*Rank 2*	*Rank 3*	*Rank 4*	*Rank 5*	TM-score range
1a	7NWW		(1-27)	0.15	0.14	0.14	0.14	0.14	0.01
1b	7OIF		(1-27)	0.14	0.15	0.17	0.15	0.15	0.03
1c	7OIG		(1-27)	0.17	0.15	0.15	0.15	0.15	0.02
2a	7OT5		**(5-70)**	0.22	0.22	0.22	0.22	0.22	0.00
2c*	1MJC		(1-70)	**0.95**	**0.95**	**0.95**	**0.95**	**0.95**	0.00

Hanazono et al. (2018)

	PDB ID	Intermediate	Modeled sequence range (starting - ending)	TM-score *Rank 1*	*Rank 2*	*Rank 3*	*Rank 4*	*Rank 5*	TM-score range
1	5ZCA		**(9-20)**	0.41	0.42	0.43	0.42	0.45	0.04
2	3WOA		**(4-45)**	**0.53**	**0.50**	**0.57**	**0.51**	0.48	0.09
3*	1LMB		(1-92)	**0.96**	**0.95**	**0.96**	**0.96**	**0.95**	0.01

Hanazono et al. (2016)

	PDB ID	Intermediate	Modeled sequence range (starting - ending)	TM-score *Rank 1*	*Rank 2*	*Rank 3*	*Rank 4*	*Rank 5*	TM-score range
1	5B3X		**(4-11)**	0.28	0.21	0.33	0.28	0.30	0.12
2	5BMY		(1-17)	0.21	0.21	0.26	0.30	0.26	0.09
4*	5B3Z		(1-35)	**0.80**	**0.81**	**0.82**	**0.77**	**0.84**	0.07

Cabrita et al. (2016)

	PDB ID	Intermediate	Modeled sequence range (starting - ending)	TM-score *Rank 1*	*Rank 2*	*Rank 3*	*Rank 4*	*Rank 5*	TM-score range
1*	6G4A		(1-114)	**0.82**	**0.81**	**0.81**	**0.81**	**0.80**	0.02
2a	2N62 (1)		(1-221)	0.47	**0.51**	**0.51**	**0.51**	**0.52**	0.05
2b	2N62 (2)		(1-221)	**0.53**	**0.56**	**0.55**	**0.58**	**0.56**	0.05
2c	2N62 (3)		(1-221)	0.45	0.44	0.44	0.44	0.44	0.01

Figure S4: The same type of results as in Fig. 4 in the main paper, except that we report TM-scores for all five top-ranked AlphaFold2-predicted structures. Specifically, the figure shows structural similarities in terms of TM-scores between Alphafold2-predicted structures and experimentally determined structures, for the 15 considered conformations of the 10 co-translational intermediates (i.e. for all but the two red ones in Fig. 3 in the main paper). For each of the four studies, there is a corresponding table; the first three table columns are already explained in Fig. 3 in the main paper (except that here we do not show or analyze an attached entity at the C-terminus). The fourth table column reports the TM-scores of all five highest-ranked predicted structures from AlphaFold2. All TM-scores with values higher than 0.50 are bolded, corresponding to structures that have the same overall fold. The fifth (last) column reports the maximum absolute TM-score difference between all pairs of the five AlphaFold2-predicted structures for a given (conformation of the) intermediate.

Agirrezabala et al. (2022)

	PDB ID	Intermediate	Modeled sequence range (starting - ending)	TM-score
1a	7NWW		(1-27)	0.15
1b	7OIF		(1-27)	0.14
1c	7OIG		(1-27)	0.17
2a	7OT5		**(5-70)**	0.22
*2c**	1MJC		(1-70)	**0.95**

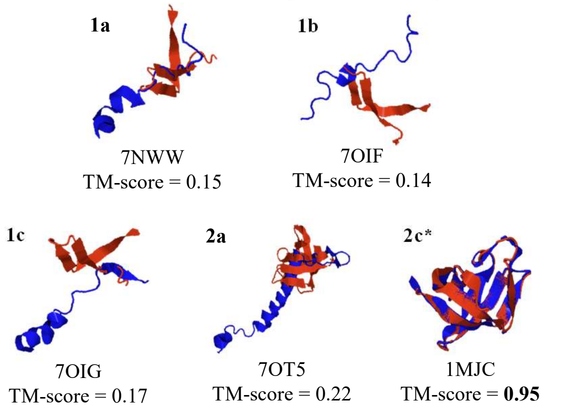

Hanazono et al. (2018)

	PDB ID	Intermediate	Modeled sequence range (starting - ending)	TM-score
1	5ZCA		**(9-20)**	0.41
2	3WOA		**(4-45)**	**0.53**
*3**	1LMB		(1-92)	**0.96**

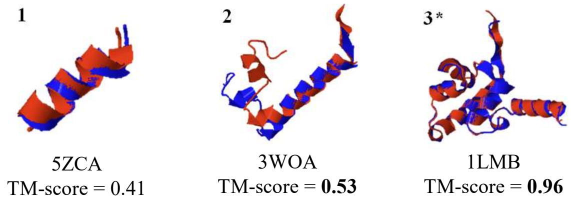

Hanazono et al. (2016)

	PDB ID	Intermediate	Modeled sequence range (starting - ending)	TM-score
1	5B3X		**(4-11)**	0.28
2	5BMY		(1-17)	0.21
*4**	5B3Z		(1-35)	**0.80**

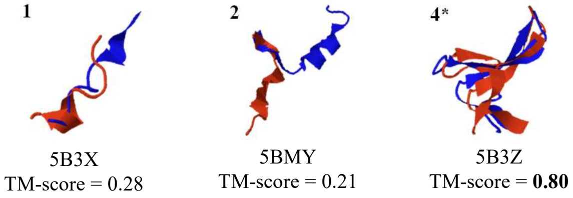

Cabrita et al. (2016)

	PDB ID	Intermediate	Modeled sequence range (starting - ending)	TM-score
*1**	6G4A		(1-114)	**0.82**
2a	2N62 (1)		(1-221)	0.47
2b	2N62 (2)		(1-221)	**0.53**
2c	2N62 (3)		(1-221)	0.45

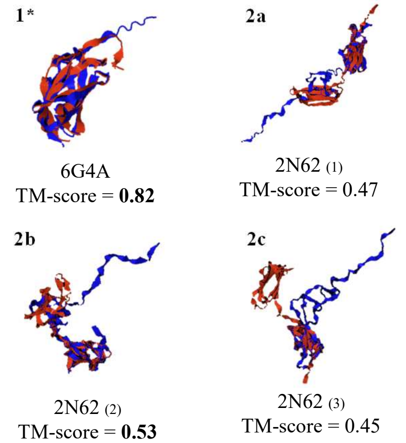

Figure S5: Structural similarities in terms of TM-scores between Alphafold2-predicted structures and experimentally determined structures for the 15 considered conformations of the 10 co-translational intermediates (i.e. for all but the two red ones in Fig. 3 in the main paper). For each of the four studies, there is a corresponding table; the first three table columns are already explained in Fig. 3 in the main paper (except that here we do not show or analyze an attached entity at the C-terminus). The fourth table column reports the TM-score of the highest-ranked predicted structure from AlphaFold2 (i.e. rank 1 from Supplementary Fig. S4). All TM-scores with values higher than 0.50 are bolded, corresponding to structures that have the same overall fold. For each TM-score, corresponding to a conformation of an intermediate, a 3D visualization of the AlphaFold2 predicted structure (blue) is overlaid with a 3D visualization of the experimental 3D structure corresponding to the modeled sequence (red).

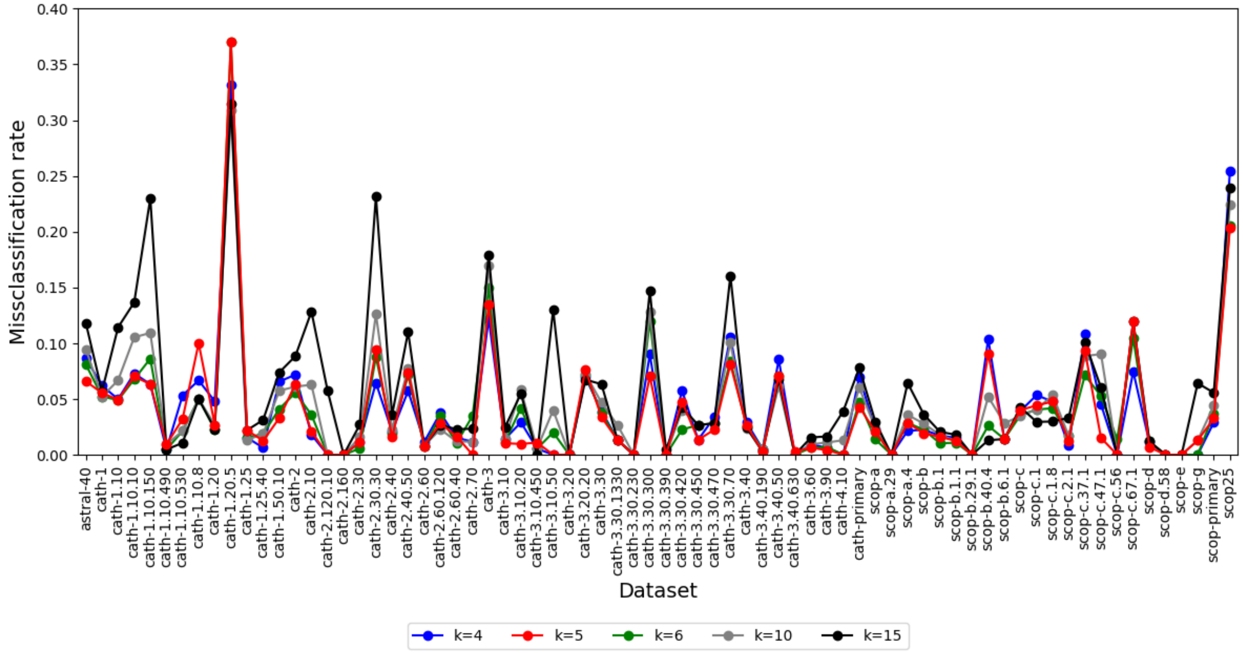

Figure S6: The effect of constructing dynamic PSNs with different values for k. As briefly mentioned in Section 3.2.2 in the main paper, dynamic PSNs were evaluated in the protein structure classification (PSC) task, specifically in the task of classifying ~44,000 protein domains with respect to their CATH (Greene et al., 2007) and SCOPe (Fox et al., 2014) structural classes. In the original dynamic PSN study (Newaz et al., 2022), the ~44,000 protein domains were organized into 72 protein domain datasets (x-axis). Also in the original study, dynamic PSNs constructed when $k = 5$ (red in the figure; Section 3.2.2 in the main paper) were evaluated in the task of PSC with respect to misclassification rate (y-axis, where lower is better). As an original contribution to our current study, we evaluate the effect on PSC misclassification rate of constructing dynamic PSNs with alternative values for k (specifically $k = 4, 6, 10, 15$, corresponding to blue, green, grey, and black in the figure, respectively). We find that out of the 72 datasets, $k = 5$ performs the best (i.e. has the lowest misclassification rate) on 34 datasets, and is within 1% misclassification rate of the best other value(s) of k on an additional 24 datasets. In other words, it is (close to) tied with, or better than, all other considered values of k on $(34 + 24)/72 = 80.6\%$ of all analyzed datasets. These findings confirm that $k = 5$ is quite a meaningful parameter choice for constructing dynamic PSNs.

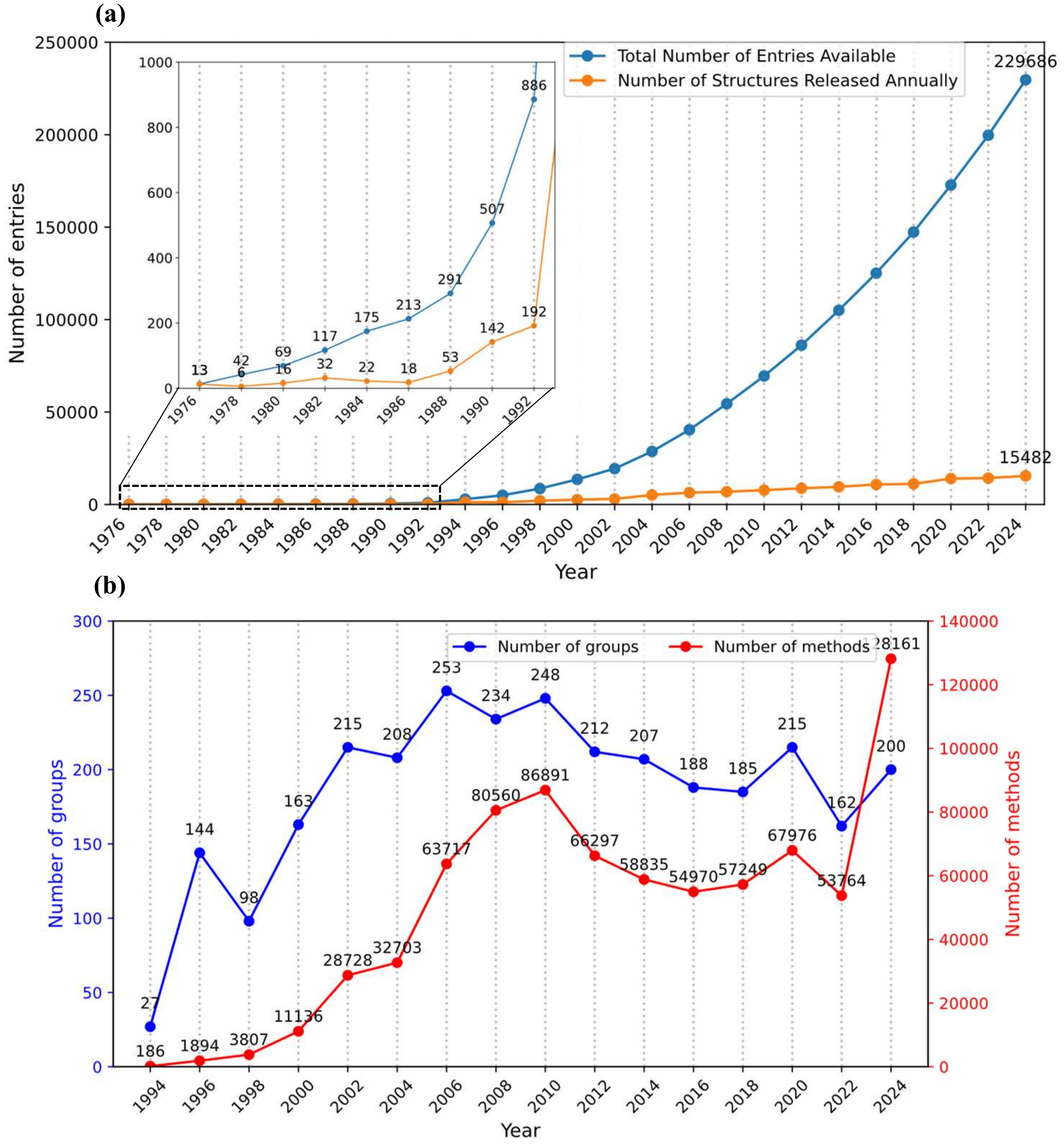

Figure S7: The growth of **(a)** available protein 3D structures (i.e. entries) in PDB over time and **(b)** participating research groups and submitted computational predictive methods (also referred to as "models" in some literature) in the CASP competition over time. We collected these statistics from `https://www.rcsb.org/stats` and `https://predictioncenter.org`, respectively.

	AlphaFold 2	RoseTTAFold	trRosetta	RaptorX	DMPfold	EVfold	SAINT2	Rosetta	Length
**10 decoys**									
Unsupervised accuracy	—	0.614	0.614	0.560	0.565	0.552	0.554	0.552	—
Unsupervised F1-score	—	0.637	0.588	0.472	0.679	0.525	0.586	0.513	—
Supervised accuracy	—	0.607	0.576	0.551	0.588	0.568	0.538	0.527	**0.656**
Supervised F1-score	—	0.637	0.620	0.558	0.667	0.643	0.620	0.655	**0.731**
AUROC	—	0.675	0.654	0.626	0.594	0.605	0.608	0.560	**0.739**
**200 decoys**									
Unsupervised accuracy	—	0.623	0.546	0.576	0.556	0.608	—	—	—
Unsupervised F1-score	—	0.663	0.638	0.610	0.687	0.616	—	—	—
Supervised accuracy	—	0.612	0.573	0.563	0.581	0.610	—	—	**0.656**
Supervised F1-score	0.613 *	0.649	0.640	0.565	0.667	0.645	—	—	**0.731**
AUROC	0.591 *	0.669	0.631	0.602	0.622	0.658	—	—	**0.739**

Table S1: Results from the Outeiral et al. (2022) study on the performance of their eight considered protein structure prediction methods and the chain length ("Length") method as a basic benchmark approach, in their first considered task of examining whether the predicted post-translational pathways predict proteins' folding kinetics classes. The two blue rows correspond to the number of predicted post-translational pathways (i.e., decoys) generated per protein by each method. The remaining rows contain the performance results for the supervised and unsupervised aspect of the considered task, with respect to three evaluation measures (accuracy, F1-score, and AUROC). Columns represent the 8 + 1 considered methods. In a given row, the bolded value indicates the best performing method. Performance scores that have an asterisk indicate that the score was computed with respect to only one decoy, rather than 10 or 200 decoys.

References

Agirrezabala, X., Samatova, E., Macher, M., Liutkute, M., Maiti, M., Gil-Carton, D., Novacek, J., Valle, M., and Rodnina, M.V. (2022). A switch from α-helical to β-strand conformation during co-translational protein folding. *The EMBO Journal*, 41(4):e109175.

Baek, M., DiMaio, F., Anishchenko, I., Dauparas, J., Ovchinnikov, S., Lee, G.R., Wang, J., Cong, Q., Kinch, L.N., Schaeffer, R.D., et al. (2021). Accurate prediction of protein structures and interactions using a three-track neural network. *Science*, 373(6557):871–876.

Cabrita, L.D., Cassaignau, A.M., Launay, H.M., Waudby, C.A., Wlodarski, T., Camilloni, C., Karyadi, M.E., Robertson, A.L., Wang, X., Wentink, A.S., et al. (2016). A structural ensemble of a ribosome–nascent chain complex during cotranslational protein folding. *Nature Structural & Molecular Biology*, 23 (4):278–285.

de Oliveira, S.H., Law, E.C., Shi, J., and Deane, C.M. (2018). Sequential search leads to faster, more efficient fragment-based de novo protein structure prediction. *Bioinformatics*, 34(7):1132–1140.

Farías-Rico, J.A., Ruud Selin, F., Myronidi, I., Frühauf, M., and Von Heijne, G. (2018). Effects of protein size, thermodynamic stability, and net charge on cotranslational folding on the ribosome. *Proceedings of the National Academy of Sciences*, 115(40):E9280–E9287.

Fox, N.K., Brenner, S.E., and Chandonia, J.M. (2014). SCOPe: Structural Classification of Proteins—extended, integrating SCOP and ASTRAL data and classification of new structures. *Nucleic Acids Research*, 42(D1):D304–D309.

Greene, L.H., Lewis, T.E., Addou, S., Cuff, A., Dallman, T., Dibley, M., Redfern, O., Pearl, F., Nambudiry, R., Reid, A., et al. (2007). The CATH domain structure database: new protocols and classification levels give a more comprehensive resource for exploring evolution. *Nucleic Acids Research*, 35(suppl_1): D291–D297.

Greener, J.G., Kandathil, S.M., and Jones, D.T. (2019). Deep learning extends de novo protein modelling coverage of genomes using iteratively predicted structural constraints. *Nature Communications*, 10(1): 3977.

Hanazono, Y., Takeda, K., and Miki, K. (2016). Structural studies of the n-terminal fragments of the WW domain: Insights into co-translational folding of a beta-sheet protein. *Scientific Reports*, 6(1):34654.

Hanazono, Y., Takeda, K., and Miki, K. (2018). Co-translational folding of α-helical proteins: structural studies of intermediate-length variants of the λ repressor. *FEBS Open Bio*, 8(8):1312–1321.

Jumper, J.M., Evans, R., Pritzel, A., Green, T., Figurnov, M., Ronneberger, O., Tunyasuvunakool, K., Bates, R., Žídek, A., Potapenko, A., Bridgland, A., Meyer, C., Kohl, S.A.A., Ballard, A., Cowie, A., Romera-Paredes, B., Nikolov, S., Jain, R., Adler, J., Back, T., Petersen, S., Reiman, D., Clancy, E., Zielinski, M., Steinegger, M., Pacholska, M., Berghammer, T., Bodenstein, S., Silver, D., Vinyals, O., Senior, A.W., Kavukcuoglu, K., Kohli, P., and Hassabis, D. (2021). Highly accurate protein structure prediction with AlphaFold. *Nature*, 596:583 – 589.

Kelkar, D.A., Khushoo, A., Yang, Z., and Skach, W.R. (2012). Kinetic analysis of ribosome-bound fluorescent proteins reveals an early, stable, cotranslational folding intermediate. *Journal of Biological Chemistry*, 287 (4):2568–2578.

Mariani, V., Biasini, M., Barbato, A., and Schwede, T. (2013). lDDT: a local superposition-free score for comparing protein structures and models using distance difference tests. *Bioinformatics*, 29(21):2722–2728.

Marks, D.S., Colwell, L.J., Sheridan, R., Hopf, T.A., Pagnani, A., Zecchina, R., and Sander, C. (2011). Protein 3D structure computed from evolutionary sequence variation. *PLOS One*, 6(12):e28766.

Neudecker, P., Robustelli, P., Cavalli, A., Walsh, P., Lundström, P., Zarrine-Afsar, A., Sharpe, S., Vendruscolo, M., and Kay, L.E. (2012). Structure of an intermediate state in protein folding and aggregation. *Science*, 336(6079):362–366.

Newaz, K., Piland, J., Clark, P.L., Emrich, S.J., Li, J., and Milenković, T. (2022). Multi-layer sequential network analysis improves protein 3D structural classification. *Proteins: Structure, Function, and Bioinformatics*, 90(9):1721–1731.

Olechnovič, K., Monastyrskyy, B., Kryshtafovych, A., and Venclovas, Č. (2019). Comparative analysis of methods for evaluation of protein models against native structures. *Bioinformatics*, 35(6):937–944.

Outeiral, C., Nissley, D.A., and Deane, C.M. (2022). Current structure predictors are not learning the physics of protein folding. *Bioinformatics*, 38:1881–1887.

Peng, J. and Xu, J. (2011). RaptorX: exploiting structure information for protein alignment by statistical inference. *Proteins: Structure, Function, and Bioinformatics*, 79(S10):161–171.

Samelson, A.J., Bolin, E., Costello, S.M., Sharma, A.K., O'Brien, E.P., and Marqusee, S. (2018). Kinetic and structural comparison of a protein's cotranslational folding and refolding pathways. *Science Advances*, 4(5):eaas9098.

Schaap, M.G., Leij, F.J., and Van Genuchten, M.T. (2001). Rosetta: A computer program for estimating soil hydraulic parameters with hierarchical pedotransfer functions. *Journal of Hydrology*, 251(3-4):163–176.

Xu, J. and Zhang, Y. (2010). How significant is a protein structure similarity with TM-score= 0.5? *Bioinformatics*, 26(7):889–895.

Yang, J., Anishchenko, I., Park, H., Peng, Z., Ovchinnikov, S., and Baker, D. (2020). Improved protein structure prediction using predicted interresidue orientations. *Proceedings of the National Academy of Sciences*, 117(3):1496–1503.

Zhang, Y. and Skolnick, J. (2004). Scoring function for automated assessment of protein structure template quality. *Proteins: Structure, Function, and Bioinformatics*, 57(4):702–710.

Zhou, Z., Feng, H., Ghirlando, R., and Bai, Y. (2008). The high-resolution NMR structure of the early folding intermediate of the Thermus thermophilus ribonuclease H. *Journal of Molecular Biology*, 384(2): 531–539.
